# Understanding the Relationship Between Avoidant/Restrictive Food Intake Disorder and Obsessive–Compulsive Symptoms: A Systematic Review

**DOI:** 10.3390/nu18050874

**Published:** 2026-03-09

**Authors:** Michelangelo Di Luzio, Valeria Villani, Giulia D’Amario, Ilaria Bertoncini, Alessandra Minutolo, Valeria Zanna, Stefano Vicari, Maria Pontillo

**Affiliations:** 1 Child and Adolescent Neuropsychiatry Unit, Bambino Gesù Children’s Hospital, IRCCS, Viale Ferdinando Baldelli, 41, 00146 Rome, Italy; valeriavillani@icloud.com (V.V.); giulia.damario@opbg.net (G.D.); ilaria.bertoncini@opbg.net (I.B.); minutolo.alessandra@gmail.com (A.M.); valeria.zanna@opbg.net (V.Z.); stefano.vicari@opbg.net (S.V.); maria.pontillo@opbg.net (M.P.); 2 Department of Neuroscience, Catholic University of the Sacred Heart, 00168 Rome, Italy; 3 Life Sciences and Public Health Department, Catholic University of the Sacred Heart, 00168 Rome, Italy

**Keywords:** avoidant/restrictive food intake disorder, obsessive–compulsive disorder, ARFID, OCD, eating disorders, comorbidity, clinical features

## Abstract

**Background:** It is well documented in the scientific literature that obsessive–compulsive disorder (OCD) and various eating disorders may present overlapping psychopathological traits. Exploring these aspects could help to identify underlying features that connect different diagnostic categories. However, evidence is lacking regarding certain less-studied eating disorders, such as avoidant/restrictive food intake disorder (ARFID). The aim of this review is to investigate the presence of comorbidity between OCD and ARFID and, consequently, the psychopathological similarities between these disorders. **Methods:** A systematic review of the PubMed/MEDLINE, Scopus and PsycInfo databases was conducted. To ensure methodological rigor, the review process followed the PRISMA (Preferred Reporting Items for Systematic Reviews and Meta-Analyses) recommendations. **Results:** After removing duplicates and applying the exclusion criteria, nine studies were included. Results indicated that although ARFID presents with primarily food-related symptomatology, the two disorders demonstrate both overlapping and distinct psychopathological characteristics. A tendency toward comorbidity is evident; however, symptom presentation appears to be influenced by age. Specifically, lower obsessive–compulsive comorbidity is observed during childhood and adolescence. Nevertheless, OCD in younger populations is more frequently associated with a fear-driven ARFID profile compared to older individuals. The co-occurrence of these conditions complicates treatment; OCD-related symptoms often show limited responsiveness to conventional approaches used for eating disorders. **Conclusions**: ARFID and OCD share partially overlapping psychopathological features, with comorbidity patterns varying by age. Recognizing these shared and disorder-specific traits—and investigating them through longitudinal studies—may guide more targeted, personalized interventions and improve treatment outcomes.

## 1. Introduction

### Diagnostic Background and Rationale

The conceptualization of eating disorders (EDs) has significantly expanded over the past decade. The recent inclusion of Avoidant/Restrictive Food Intake Disorder (ARFID) as a distinct diagnosis in the Diagnostic and Statistical Manual of Mental Disorders 5th Edition (DSM-5) is one such development [1]. Given its recent characterization, further research into the profile of ARFID is needed [2]. While comorbidity between ARFID and conditions such as autism and anxiety disorders is well established [3,4,5,6], its association with obsessive–compulsive disorder (OCD) remains less clear. According to the DSM-5 Text Revision (DSM-5-TR) [7], some individuals diagnosed with OCD may show restrictive eating behaviors, often influenced by the sensory properties of meals or by ritualized behaviors. However, ARFID should be diagnosed as a comorbid condition only if all criteria for both disorders are met, and if restrictive eating is a prominent feature of the clinical presentation requiring specific intervention. ARFID is characterized by limited food intake and/or variety, primarily driven by cognitive and sensory factors other than body shape or weight concerns. These factors include lack of interest in food (taking small bites, excessive chewing and slow eating), avoidance based on the sensory characteristics of food (extreme sensitivity to appearance, color, odor, texture, temperature or taste) and avoidance in response to, or in anticipation of, a feared adverse experience (choking or vomiting). These presentations are not mutually exclusive and are not formally recognized as subtypes of ARFID, but rather represent distinct mechanisms observed in the disorder. In addition to its core features, ARFID is often associated with adverse consequences such as notable weight loss or stunted growth, nutritional deficiencies, reliance on nutritional supplementation, and considerable impairment in social functioning, particularly in situations involving food-related interactions [7,8,9]. ARFID was introduced in the DSM-5 in response to the clinical need to distinguish it from other EDs, as it differs in its lack of correlation with weight or body shape [10]. The diagnosis does not have an age restriction; however, it is most frequently identified in older children and younger adolescents [11,12,13]. OCD, according to the DSM-5-TR [7], is defined by the presence of persistent, intrusive thoughts (obsessions) and/or repetitive behaviors (compulsions), which are typically performed to reduce the psychological distress caused by the obsessions. Common themes of obsessions often involve the need for symmetry or precision, anxieties about illness and contamination, unacceptable sexual or religious thoughts and intrusive aggressive thoughts. Compulsive behaviors may include excessive organizing, cleaning, counting, checking, repeating routine activities, hoarding or seeking constant reassurance. Obsessive–compulsive (OC) symptoms are typically time-consuming (more than an hour per day) and interfere with functioning, particularly when there is limited awareness of the condition [14,15,16,17]. There is substantial empirical evidence supporting the comorbidity between OCD and various EDs, with anorexia nervosa (AN) and bulimia nervosa (BN) demonstrating particularly high rates of comorbidity [18]. OCD is occasionally recognized as a potential risk factor for the subsequent development of EDs [19,20]. In general, EDs accompanied by OCD tend to be associated with more severe eating symptoms and an increased likelihood of hospitalization [21,22]. The specific comorbidity data between ARFID and OCD are less well-established. Notably, ARFID was formally recognized as a distinct diagnosis only in 2013 with the publication of the DSM-5, and consequently, the literature remains limited, particularly regarding its interrelation with other psychiatric disorders. Rigidity in cognitive processing (including repetitive behaviors, perfectionism and difficulty in adapting to change or unforeseen circumstances) may contribute to the onset or persistence of certain symptoms associated with OCD and ARFID [23]. The comorbidity of OCD in patients with ARFID may complicate standard treatment approaches. Indeed, when OC symptoms are present in EDs, this may reduce the efficacy of treatments, particularly with respect to OC symptoms not related to eating symptomatology [24,25]. This necessitates tailored approaches when addressing complex conditions involving the co-occurrence of OCD and ARFID. Indeed, exploring how comorbidity arises and clarifying the connections between disorders can highlight transdiagnostic constructs that could be targeted in treatment. The aim of this review is to analyze the existing evidence regarding the comorbidity between OCD and ARFID. Particular attention is given to the differences between child/adolescent and adult populations, with a focus on clinical implications and potential directions for future research. Our primary hypothesis is that, similar to other EDs, ARFID exhibits a high comorbidity with OCD, though this may be influenced by age. Indeed, as previously observed [24], adults with EDs may show a higher prevalence of OC symptoms compared to children and adolescents. Finally, our second hypothesis posits the presence of both shared and disorder-specific psychopathological patterns, which may in turn influence treatment efficacy.

## 2. Materials and Methods

### 2.1. Literature Search

A systematic review was conducted using PubMed/MEDLINE, Scopus, and PsycInfo databases, covering the literature published up to 8 January 2026. The search utilized the following terms: “Avoidant Restrictive Food Intake Disorder” AND “Obsessive Compulsive Disorder” OR “obsessive-compulsive traits”. All authors jointly reviewed and approved the search strategy, ensuring consistency in the literature screening process.

### 2.2. Inclusion and Exclusion Criteria

Two rounds of the Delphi method were employed to develop the inclusion and exclusion criteria, which were unanimously approved by all contributing authors. To ensure methodological rigor, we structured the review process in line with the PRISMA (Preferred Reporting Items for Systematic Reviews and Meta-Analyses) guidelines [26]. Supplementary Materials include the PRISMA flowchart and checklist, along with detailed information on study outcomes and the selection process for both included and excluded studies (see Online Supplementary Material: Supplementary Figures S1 and S2, and Supplementary Table S1). The inclusion and exclusion criteria were designed to address the study’s hypotheses. Specifically, the aim was to include original studies that examined the characteristics of ARFID in relation to OCD and vice versa, or that analyzed patient populations presenting with overlapping ARFID and OCD symptomatology. To ensure the reliability of the results, potentially confounding studies were excluded, particularly those that did not provide separate data for the two disorders or did not specifically focus on this topic. Furthermore, all non-original studies on clinical populations, such as reviews, meta-analyses, and case reports, were excluded. A schematic overview of all inclusion and exclusion criteria is presented below. Studies were eligible if they met the following predefined inclusion criteria: (1) were original empirical research; (2) involved participants diagnosed with ARFID and OCD/OC traits confirmed via the Diagnostic and Statistical Manual of Mental Disorders (DSM), Third, Fourth, Fifth, or Fifth Edition, Text Revision (DSM-III/IV/5/5-TR) or International Classification of Diseases Ninth, Tenth, or Eleventh Edition (ICD-9/10/11); (3) examined clinical, genetic or neurobiological features comparing patients with ARFID and OCD/OC traits, or by assessing these features in comorbid clinical samples (i.e., patients with ARFID and OCD/OC traits, and vice versa); (4) reported separate data for ARFID and OCD. Studies that met the following exclusion criteria were removed: (1) reviews and meta-analyses (however, the reference lists of these articles were screened for additional relevant studies) (i.e., “Review”); (2) case reports and case series (i.e., “Case Report”); (3) participants not diagnosed with OCD or comorbid ARFID and OCD symptoms (i.e., “No OCD”); (4) participants not diagnosed with ARFID or comorbid OCD and ARFID symptoms (i.e., “No ARFID”); (5) did not separate data for individuals with ARFID, OCD, healthy controls or other psychiatric conditions (i.e., “Lumping”); (6) articles not relevant to the scope of the current review (i.e., “Unrelated”); (7) studies involving non-human subjects (i.e., “Animal”); (8) protocols and trials in progress; (9) corrigenda; (10) studies for which an English version was not accessible (i.e., “No English”). Any redundant records found across databases were eliminated prior to analysis.

### 2.3. Data Collection and Analysis

Extracted data were organized systematically within a structured spreadsheet template. Recorded variables included the first author, year of publication, sample size, participants’ age, sex distribution (male/female), country of origin, and study methodology—such as interviews, assessments, measurement instruments, or questionnaires employed. In addition, key findings related to the association between ARFID and OCD or OC traits were documented. The synthesis of the included studies is provided in Table 1.

### 2.4. Assessment of Risk of Bias

A bias evaluation was performed to assess the methodological quality and reliability of the included studies in accordance with the guidelines provided by the Agency for Healthcare Research and Quality (AHRQ) [27]. The AHRQ tool was selected because it is widely used and considered reliable due to its versatility and broad application in scientific literature, as it allows for a structured assessment of methodological quality in studies with different study designs. Each study was evaluated across predefined domains, including selection, performance, detection, attrition, and reporting biases. Studies were then categorized according to overall risk level (low, moderate, or high). This assessment was independently performed by the authors, with any discrepancies resolved through discussion and consensus. Full details of the bias assessment can be found in the online Supplementary Materials (see Supplementary Table S2).

## 3. Results

### 3.1. Search Outcomes

The complete search process and exclusion rationale are illustrated in the PRISMA flowchart available in the Online Supplementary Materials (Supplementary Figure S1). The database search retrieved 363 studies spanning October 1946 to December 2025. After the removal of duplicates, 351 unique citations were found. After applying the eligibility criteria, 338 studies were excluded during title/abstract screening, leaving 13 studies for full-text retrieval. Finally, nine citations were included after full-text review (see Table 1). Detailed reasons for exclusion are provided in the Online Supplementary Materials (Supplementary Table S1).

**Table 1:** Summary of included studies.

Study	Population	Design	Assessment	Results
Manwaring et al., 2024 [28]	45 patients with ARFID (males = 28, $\bar{x}$ = 26.95 ± 10.19)	Cross-sectional; United States	EPSI, EDQOL, STAI, BDI, OCI-R, PCL-5, BMI	Patients with ARFID show ↑ psychiatric symptoms, including moderate levels of depression, anxiety, and OCD. Patients also have ↑ scores on the OCI-R, indicating a moderate presence of OCD; 34% of the patients met criteria for an OCD diagnosis. ↑ OCD rates (52%) among those without a fear-based ARFID presentation.
Richson et al., 2024 [29]	686 patients with ARFID (males = 153, $\bar{x}$ = 21.62 ± 4.50); 732 patients with ARFID + Non-ARFID EDs (males = 139, $\bar{x}$ = 23.4 ± 5.15); 3239 patients with Non-ARFID EDs (males = 619, $\bar{x}$ = 24.1 ± 6.09)	Cross-sectional; United States	HMS, SCOFF, PHQ-9, GAD-7	Lifetime OCD prevalence was highest in the ARFID + Non-ARFID EDs group (39.7%), followed by ARFID only (31.1%) and Non-ARFID EDs (26.9%).
Velimirović et al., 2025 [3]	251 patients with ARFID (males = 50, $\bar{x}$ = 25 ± 8.6); 1074 patients with AN-R (males = 57, $\bar{x}$ = 25.7 ± 10.2); 597 patients with AN-BP (males = 22, $\bar{x}$ = 26.8 ± 9.8); 282 patients with BN (males = 22, $\bar{x}$ = 27.4 ± 9.4); 259 patients with BED (males = 40, $\bar{x}$ = 36.3 ± 12.4); 1267 patients with OSFED (males = 68, $\bar{x}$ = 26.7 ± 9.6)	Longitudinal; United States Treatment: individual, group, and family-based psychotherapy	OCI-R, GAD-7, PHQ-9	Patients with ARFID exhibited moderate OCD symptoms at admission, with significant post-treatment improvement (*p* < 0.001). Only 26.5% showed reliable OCD symptom reduction, which improved ↓ than anxiety and depression. In ARFID and AN-R, OCD reduction correlated with weight gain (*p* = 0.002). OCD symptoms were ↑ than depressive symptoms in ARFID. OCD showed significantly ↓ improvement compared to depression (*p* < 0.001).
Wronski et al., 2024 [30]	616 patients with ARFID (males = 375, $\bar{x}$ = 9.2 ± 1.8 years); 30.179 patients HC (males = 15.257; $\bar{x}$ = 9.3 ± 1.7 years)	Cross-sectional; Sweden	CATSSpr, NPRdc	No significant association between ARFID and OCD. Patients with ARFID had ↑ distinct psychiatric diagnoses (IRR = 4.65) and ↑ inpatient treatment days (IRR = 5.50).
Sader et al., 2023 [31]	183 patients with ARFID (males = 100, $\bar{x}$ = 10.0 ± 2.1) 2679 HC (males = 1414, $\bar{x}$ = 10.0 ± 1.9)	Cross-sectional; Netherlands	SOCS, SFQ	Results from the SOCS scale indicated that the ARFID group had slightly ↑ scores for obsessive symptoms compared to HC (*p* < 0.05).
Kambanis et al., 2020 [32]	74 patients with ARFID (males = 74, $\bar{x}$ = 15.0 ± 3.5)	Cross-sectional; United States	K-SADS, EDA-5; PARDI	OCD is diagnosed in 4% of patients. ARFID patients with ↑ levels of sensory sensitivity are significantly more likely to have or develop OCD (*p* = 0.003). Similarly, a strong fear of negative consequences in ARFID patients increases the risk of having or developing OCD (*p* = 0.003). In contrast, lack of interest in food in ARFID EDs was not significantly associated with OCD (*p* = 0.188).
Zickgraf et al., 2019 [33]	22 patients with ARFID (males = 18, $\bar{x}$ = 11.23 ± 5.76)	Cross-sectional; United States	K-SADS, ADIS, ADSI	68.2% of the sample exhibited psychiatric comorbidities, including anxiety disorders (54.5%), OCD (13.6%), tic disorders (9.1%), and ADHD (13.6%).
Bryson et al., 2018 [34]	20 patients with ARFID (males = 6, $\bar{x}$ = 14.12 ± 1.48); 42 patients with AN (males = 2, $\bar{x}$ = 11.43 ± 1.55)	Cross-sectional; United States	RAD, ChEAT	ARFID and AN groups show similar scores (*p* = 0.33) for OC symptomatology.
Zickgraf et al., 2016 [35]	46 patients with ARFID (males = 11); 133 patients Picky eaters (males = 60); 38 patients with ED attitudes (males = 9); 189 typical eaters (males = 104) MTurk sample: $\bar{x}$ = 33.92 ± 10.54 Support group sample: $\bar{x}$ = 40.42 ± 13.31	Cross-sectional; United States	OCI-R, EAT-26, DASS-21, FNS, Inflexibility Index, Sensory Sensitivity Scale, ARFID Symptom Checklist	The ARFID group exhibited significantly ↑ OCD symptoms compared to typical eaters (*p* < 0.001) and picky eaters without ARFID (*p* < 0.05), but showed no significant difference compared to those with ED attitudes.

Abbreviations: ADIS, Anxiety Disorders Interview Schedule; ADSI, ARFID Diagnostic and Severity Interview for children; AN, Anorexia Nervosa; AN-R, Anorexia Nervosa, Restricting Type; AN-BP, Anorexia Nervosa, Binge–Purge type; BDI, Beck Depression Inventory; BED, Binge-Eating Disorder; BN, Bulimia Nervosa; CATSSpr, Child and Adolescent Twin Study in Sweden parent report; ChEAT, Children’s Eating Attitudes Test; DASS-21, Depression Anxiety Stress Scales–21; EAT-26, The Eating Attitudes Test-26; ED, eating disorder; EDA-5, Eating Disorder Assessment for DSM-5; EDQOL, Eating Disorder Quality of Life; EPSI, Eating Pathology Symptoms Inventory; FNS, Food Neophobia Scale; GAD-7, 7-item Generalized Anxiety Disorder; HC, healthy control; HMS, Healthy Minds Study; IRR, incidence rate ratio; K-SADS, Kiddie Schizophrenia and Affective Disorders Schedule, NPRdc, National Patient Register diagnostic codes; OC, obsessive–compulsive; OCI-R, Obsessive-Compulsive Inventory–Revised; OSFED, Other Specified Feeding or Eating Disorders; PARDI, Pica, ARFID and Rumination Disorder Interview; PCL-5, Post Traumatic Posttraumatic Stress Disorder Checklist for DSM-5; PHQ-9, Patient Health Questionnaire-9; RAD, retrospective assessment of diagnosis; SCOFF, SCOFF questionnaire; SFQ, Stanford Feeding Questionnaire; SOCS, Short OCD Screener; STAI, State-Trait Anxiety Inventory; $\bar{x}$ , mean age; ↑, higher/more/increased; ↓, lower/lesser/decreased.

### 3.2. Characteristics of Included Studies

In this first section, we present methodological aspects of each included study, aiming to highlight both commonalities and divergences across studies. The main characteristics—such as sample composition and size, study design, and assessment methods used—are reported for each study in Table 1.

The included studies were evenly divided by participant age: two studies involved adult populations [29,35], two studies included a mixed-age sample of adults and older adolescents [3,28], and five focused on children and adolescents [30,31,32,33,34]. Sex distribution appears to be consistent across all studies. In general, the studies involving children included smaller samples, which may limit the reliability of their findings. In contrast, studies on adults tend to involve larger samples, likely reflecting the relative ease of conducting research in adult compared to pediatric populations.

Eight of the studies employed cross-sectional designs, while only the study by Velimirović et al. [3] was a longitudinal cohort study conducted in an adult sample. Nearly all studies focused on outpatient populations, only Manwaring et al. exclusively examined inpatients [28], while both Bryson et al. and Velimirović et al. included mixed samples of inpatients and outpatients [3,34]. Notably, Bryson et al. [34] included participants in a partial hospitalization treatment setting.

While most studies established the diagnosis of ARFID through in-person clinical evaluation, a few relied on alternative assessment methods. Richson et al. relied on self-reported diagnoses [29]; Wronski et al. [30] used national registry data based on parent-reported diagnosis; and Bryson et al. conducted a retrospective diagnostic analysis [34]. The measures used for the assessment of OC symptoms vary considerably across studies. Richson et al. [29], Wronski et al. [30] and Bryson et al. [34] respectively used the same assessment procedure used for the diagnosis of ARFID. Many studies used self-report questionnaires (OCI-R; SOCS). The OCI-R was the most frequently used tool [3,28,35]. While widely adopted, it should be noted that the OCI-R is a self-report measure and is therefore subject to reporting bias. Other studies used semi-structured diagnostic interviews such as the K-SADS, which provide higher diagnostic reliability and validity [32,33]. This variability may pose challenges to the comparability of findings. Nonetheless, the research objectives across studies appear relatively consistent, contributing to the overall coherence of the findings.

### 3.3. Main Findings

This review includes studies that primarily address the clinical aspects of ARFID and OCD. The majority of studies focused on OC symptomatology within ARFID groups, whereas none examined the presence of ARFID-related symptoms in OCD samples. Additionally, the underlying psychopathological features that may support the comorbidity between ARFID and OCD were explored, as well as the differential response to specific psychotherapeutic treatments. In the following section, we present the main findings of the included studies, considering them collectively and highlighting both commonalities and divergences. We report the findings for each study in Table 1 and specifically for the first two paragraphs in Table 2.

#### 3.3.1. Findings on Comorbidity Between ARFID and OCD

The comorbidity between ARFID and OCD has been investigated in several studies. Patients with ARFID may exhibit symptoms associated with OCD. However, the nature and strength of this comorbidity appear to change depending on age (Table 2). Studies conducted among adults suggest that ARFID is frequently associated with moderate to severe OCD symptoms [3,28,29,35]. These findings indicate that the relationship between ARFID and OCD may be particularly pronounced in adults, potentially exacerbating the clinical presentation of both conditions. In contrast, in pediatric populations, the link between ARFID and OCD appears to be weaker [30,31,32,33]. These data may suggest that ARFID in younger subjects may manifest with fewer OCD symptoms compared to adults. However, these results must also be interpreted in light of the methodological quality of the included studies (Table 2). In fact, some adult-focused studies have methodological limitations that may impact their findings [28,29]. At the same time, Wronski et al. [30], who did not identify a significant comorbidity between ARFID and OCD in a child population, had a study with a moderate risk of bias.

In general, both in adults and in children/adolescents, ARFID appears to exhibit a comorbidity with OCD that is comparable to that observed in other EDs [3,29,34,35]. Specifically, it seems to be similar in terms of comorbidity rates and symptom severity to those observed in AN [3,34] (Table 2). Therefore, our initial hypothesis appears to be supported.

#### 3.3.2. OCD and ARFID Psychopathological Profiles

Two studies have explored how comorbid presentations with OCD vary according to the psychopathological characteristics of ARFID [28,32]. Specifically, these studies have investigated how comorbidity patterns differ depending on which of the three primary drivers of food avoidance is predominant: lack of interest in eating, fear of aversive consequences, or heightened sensory sensitivity (Table 2). The studies have yielded partially convergent results identifying sensory hypersensitivity as a psychopathological feature potentially associated with the presence or development of OC symptoms. At the same time, findings differ markedly regarding the relationship between OCD and the “fear of aversive consequences” ARFID profile. Differences in age composition of the two samples may have contributed to the divergence in results (Table 2). It could be hypothesized that, in younger patients, the fear-based profile is more strongly associated with OC symptoms than in older adolescents and adults, among whom profiles lacking fear-based aspects show higher comorbidity with OCD. Additionally, the “heightened sensory sensitivity” profile is associated with OCD independently of age.

**Table 2:** Summary of Findings on the Comorbidity Between ARFID and OCD.

Study	Age	ARFID Diagnosis	OCD assessment	ARFID-OCD Comorbidity	Risk of Bias	Key Limitations
Manwaring et al. [28]	Adults and adolescents	DSM-5-based clinical interview	Self-report questionnaire (OCI-R)	Moderate prevalence (34%). Lower prevalence in fear-based profile (17.39%) and higher among those without (52.38%)	Low	Small sample; self-report OCD assessment
Richson et al. [29]	Adults	Self-report questionnaire (survey)	Self-report questionnaire (survey)	Significantly higher lifetime prevalence in ARFID + non-ARFID EDs group compared to non-ARFID EDs only group	Moderate	Large sample; Questionnaire-based sample selection; small effects in statistical analysis
Zickgraf et al. [35]	Adults	Author-developed self-report questionnaire based on DSM-5 criteria	Self-report questionnaire (OCI-R)	Significant presence and similar to other EDs	Low	Self-report measures for diagnosis and assessment; demographically different samples
Velimirović et al., 2025 [3]	Adults and adolescents	DSM-5-based clinical interview	Self-report questionnaire (OCI-R)	More pronounced and more similar to AN-R than to other EDs	Low	Self-report OCD assessment
Sader et al. [31]	Children	DSM-5-based clinical interview	Self-report questionnaire (SOCS)	Low prevalence	Low	Self-report OCD assessment
Kambanis et al. [32]	Children and adolescents	Semi-structured interview (EDA-5; K-SADS)	Semi-structured interview (K-SADS)	Low current and lifetime prevalence (4%). Higher current and lifetime prevalence in the fear-based and sensory hypersensitivity profiles. No significant association with the lack of interest profile	Low	Small sample; reliable tools for diagnosis and assessment
Zickgraf et al. [33]	Children and adolescents	Semi-structured interview (K-SADS; ADSI)	Semi-structured interview (K-SADS)	Low prevalence (13%)	Low	Small sample; reliable tools for diagnosis and assessment
Wronski et al. [30]	Children	Parent reports	Parent reports	No significant association	Moderate	Retrospective recruitment; parent-reported diagnosis and assessment
Bryson et al., 2018 [34]	Children and adolescents	Retrospective chart review	Retrospective chart review	Similar to AN	Moderate	Small sample; retrospective methodology

Abbreviations: ADSI, ARFID Diagnostic and Severity Interview for children; AN, Anorexia Nervosa; AN-R, Anorexia Nervosa, Restricting Type; ARFID, Avoidant/Restrictive Food Intake Disorder; DSM-5, Diagnostic and Statistical Manual of Mental Disorders, Fifth Edition; ED, eating disorder; EDA-5, Eating Disorder Assessment for DSM-5; K-SADS, Kiddie Schizophrenia and Affective Disorders Schedule; OCD, Obsessive–Compulsive Disorder; OCI-R, Obsessive–Compulsive Inventory—Revised; SOCS, Short OCD Screener.

#### 3.3.3. OCD, ARFID and Psychotherapeutic Treatments

Velimirovic et al. [3] investigated the effectiveness of intensive psychotherapeutic treatments in adult inpatients and outpatients with ARFID and comorbid anxiety, depression, and OCD. The interventions were primarily focused on ARFID but also included exposure and response prevention (ERP) sessions. ARFID symptoms improved significantly across all outcome measures. The findings suggest that ED treatments can also produce meaningful reductions in co-occurring psychiatric symptoms, including OCD. However, the data also indicate that OCD symptoms may be less responsive to treatment compared to anxiety or depression: only 26.5% of the ARFID group showed a reliable reduction in OCD symptoms. Although this is the only study included that evaluated treatment efficacy in comorbid ARFID cases, it represents one of the methodologically strongest investigations, given its large sample size and longitudinal cohort design. Moreover, the results are particularly noteworthy as they point to the potential need for more tailored interventions in the treatment of EDs.

Support for our second hypothesis comes from results indicating that the presentation of OC symptoms in ARFID may be influenced by specific psychopathological mechanisms underlying food avoidance, which may be partially age-dependent. Moreover, the presence of both overlapping and divergent features is reflected in the partial efficacy of psychotherapeutic interventions in ARFID patients with comorbid OCD.

## 4. Discussion

### 4.1. Comorbidity and Psychopathological Profiles

The relationship between ARFID and OCD remains an emerging area of investigation, with increasing evidence suggesting a potential overlap in symptomatology. This review highlights the complexity of this relationship. Our two hypotheses, based on findings from our previous studies, appear to be supported.

In a previous study conducted by our group, OCD symptoms during childhood and adolescence were found to be more strongly associated with the binge–purge subtype of EDs than with the restrictive subtype [24]. In adulthood, however, this distinction appears to diminish and may be attributable to a general age-related increase in comorbidity between EDs and OCD [36,37,38]. From this perspective, the findings of the present review are consistent with previous evidence if ARFID is conceptualized as a restrictive-type ED. Accordingly, both restrictive EDs and ARFID seem to exhibit lower levels of comorbidity with OCD in childhood and adolescence, but higher levels in adulthood.

Regarding psychopathological features, the included studies underscore a complex and heterogeneous relationship between ARFID and OC symptoms across developmental age and adulthood. Children and adolescents could present lower OCD comorbidity, but with a stronger association with the “fear of aversive consequences” profile compared to late adolescents and adults. Cognitive-behavioral frameworks conceptualize obsessions as fear-inducing thoughts or images that foster an inflated sense of responsibility for preventing the feared outcome, thereby increasing distress and prompting neutralizing behaviors to avert the perceived threat [39]. A similar mechanism may also underlie the ARFID subtype characterized by food avoidance driven by fear of adverse consequences, such as choking or vomiting. One possible explanation for the stronger representation of fear-based ARFID in childhood may relate to developmental differences in fear processing and avoidance learning. In the pediatric population, fear responses are often closely and directly linked to avoidance behaviors, with limited cognitive mediation [40]. Moreover, research in pediatric OCD has highlighted the central role of fear-driven symptom dimensions in children and adolescents [39]. At the same time, the existing literature indicates that OC symptoms are linked to tactile and oral sensory hypersensitivity in both children and adults with OCD [41,42,43]. Similarly, ARFID—both in pediatric and adult populations—has been associated with sensory hypersensitivity [44,45]. Taken together, these findings suggest that sensory hypersensitivity may represent a transdiagnostic feature common to both disorders across developmental stages, whereas fear-based symptoms appear to be more age-specific.

### 4.2. Treatment Implications

The combination of ARFID and OCD can make psychotherapeutic treatment particularly challenging. This complexity reflects the divergence in therapeutic targets between the two conditions. Although therapeutic protocols, particularly those based on cognitive-behavioral therapy (CBT), have been developed over time specifically for either OCD or EDs, there are currently no treatment programs designed to address both symptom domains simultaneously. Research in the treatment of EDs indicates that while CBT is effective in improving eating-related symptoms in individuals with EDs and OC traits, it does not comprehensively address non-eating-related OC features, which may persist despite improvements in eating behaviors [25]. This suggests that a standard CBT protocol for EDs may not be sufficient for individuals with comorbid OCD symptoms, necessitating adjunctive interventions tailored to OC symptomatology. As observed in our review, although OCD symptoms in ARFID may improve in response to intensive treatment for EDs, sometimes even more so than in other EDs, the rate of improvement (of OCD symptoms) remains lower than that observed for other comorbidities, such as depression or anxiety [3]. The co-occurrence of EDs and OCD is indeed considered a negative prognostic factor and is associated with a poorer outcome [21,22]. In this context, ERP, the first-line intervention for OCD [46], has shown promise in reducing compulsive behaviors in individuals presenting with both ED symptoms and OC features [47]. Moreover, some findings suggest that integrating ERP into CBT-based interventions for EDs, particularly in cases where rigid food rules and ritualistic behaviors resemble classic OCD patterns, may enhance treatment outcomes [48]. Considering the findings on comorbidity, this may be particularly true for adult patients with ARFID. The inclusion of ERP sessions in the study by Velimirovic et al. [3] may account for the partial improvement observed. Further specific studies on the treatment of OCD symptoms in individuals with ARFID, particularly regarding CBT-ERP integration, are needed to clarify this issue.

Beyond treatment integration, the present findings also suggest practical implications. Assessment of OC symptoms may be incorporated into the clinical evaluation of individuals with ARFID. While the available evidence remains limited, early recognition of overlapping symptom dimensions could support more informed treatment planning and help prevent the persistence of comorbid features over time.

### 4.3. Limitations and Strengths

The findings of this review should be interpreted in light of the considerable heterogeneity across the included studies. Variability in sample characteristics, age composition, study design, and diagnostic procedures for both ARFID and OCD may have influenced the reported comorbidity rates. Such clinical and methodological heterogeneity limits direct comparability across studies. At the same time, this diversity represents a potential strength, as it provides a broader and more ecologically valid overview of the ARFID–OCD association across different populations and research contexts. Indeed, one of its strengths is the inclusion of both children and adults, allowing for a broader understanding of how this comorbidity manifests across the lifespan. However, there are some limitations to consider. One notable limitation is the reliance on self-report measures in certain studies, which can introduce bias due to subjective reporting and potential misinterpretation of symptoms by participants. Another limitation concerns the small sample sizes in some of the included studies; this can affect the generalizability of the results and reduce statistical power. Additionally, the cross-sectional nature of most studies included in the review precludes causal inferences. Collectively, these methodological characteristics contribute to variability in the risk of bias among the included studies. The studies show a generally low level of overall risk of bias, with the exception of Wronski et al. [30], Bryson et al. [34] and Richson et al. [29], which present a moderate risk. The moderate risk is primarily attributable to the tools used for ARFID diagnosis and OCD assessment: retrospective methodology and parent-report assessments in Wronski et al. [30], a self-report questionnaire in Bryson et al. [34] and self-report assessments in Richson et al. [29] (Table 2 and Supplementary Table S2). A further limitation concerns the search strategy, which prioritized specificity over sensitivity. Although broader terms might have increased study retrieval, they would likely have yielded a large number of non-specific records due to the overlap between ARFID and other EDs; however, this approach may have limited the comprehensiveness of the search.

## 5. Conclusions

This review highlights shared psychopathological features between ARFID and OCD, while emphasizing relevant developmental differences. Although a general tendency toward comorbidity is evident, age appears to influence its manifestation: children and adolescents with ARFID tend to have lower overall rates of OCD comorbidity; however, in this group, the fear-driven profile is more strongly associated with OC symptoms than in older patients. From a therapeutic perspective, integrating ERP into CBT protocols for EDs may enhance treatment outcomes in patients presenting with comorbid features. Overall, these findings suggest that ARFID and OCD may lie along a shared transdiagnostic continuum that evolves across development, with age shaping both comorbidity rates and the expression of shared psychopathological features. In conclusion, there is a clear need for further research to clarify the developmental trajectories of ARFID and OCD symptoms, particularly through longitudinal designs capable of elucidating their temporal relationship and identifying potential risk factors for co-occurrence. Future studies should prioritize larger samples to enhance external validity and should incorporate multi-method assessment strategies—including clinician-rated evaluations and neurocognitive testing—to strengthen the reliability and precision of findings. Moreover, research on treatment outcomes across different age groups is warranted to determine whether developmental differences influence therapeutic response and to guide more targeted and age-sensitive interventions.

## Data Availability

No datasets were generated or analyzed during the current study.

## Supplementary Materials

The following supporting information can be downloaded at: https://www.mdpi.com/article/10.3390/nu18050874/s1, Figure S1: PRISMA flowchart; Figure S2. PRISMA Checklist; Table S1: Table of Included/excluded studies; Table S2: Risk of bias assessment.

## Abbreviations

The following abbreviations are used in this manuscript:

ADHD	Attention-Deficit/Hyperactivity Disorder
ADSI	ARFID Diagnostic and Severity Interview
AHRQ	Agency for Healthcare Research and Quality
AN	Anorexia Nervosa
AN-R	Anorexia Nervosa—Restrictive Type
ASD	Autism Spectrum Disorder
CBT	Cognitive Behavioral Therapy
DSM-5-TR	Diagnostic and Statistical Manual of Mental Disorders, Fifth Edition, Text Revision
ED	Eating Disorder
ERP	Exposure and Response Prevention
GAD	Generalized Anxiety Disorder
K-SADS	Kiddie Schedule for Affective Disorders and Schizophrenia
OCI-R	Obsessive–Compulsive Inventory—Revised
OCD	Obsessive–Compulsive Disorder
OC	Obsessive Compulsions
PRISMA	Preferred Reporting Items for Systematic Reviews and Meta-Analyses
RAD	Retrospective Assessment of Diagnosis
SOCS	Short OCD Screener
