# Supplementary material for: Understanding the Relationship Between Avoidant/Restrictive Food Intake Disorder and Obsessive–Compulsive Symptoms: A Systematic Review
Source: Nutrients. 2026 Mar 9;18(5):874. doi: 10.3390/nu18050874 (PMC12987355; doi:10.3390/nu18050874)
Supplement: Supplementary file 1 [file nutrients-18-00874-s001.zip › nutrients-4125585-supplementary.pdf]

Supplementary Materials

Supplementary Figure S1. PRISMA flowchart.

Search strategy:
PubMed: "Avoidant Restrictive Food Intake Disorder" AND "Obsessive Compulsive Disorder" OR "obsessive-compulsive traits"
PsycInfo: "Avoidant Restrictive Food Intake Disorder" AND "Obsessive Compulsive Disorder" OR "obsessive-compulsive traits"
Scopus: "Avoidant Restrictive Food Intake Disorder" AND "Obsessive Compulsive Disorder" OR "obsessive-compulsive traits"
on 08-01-2026

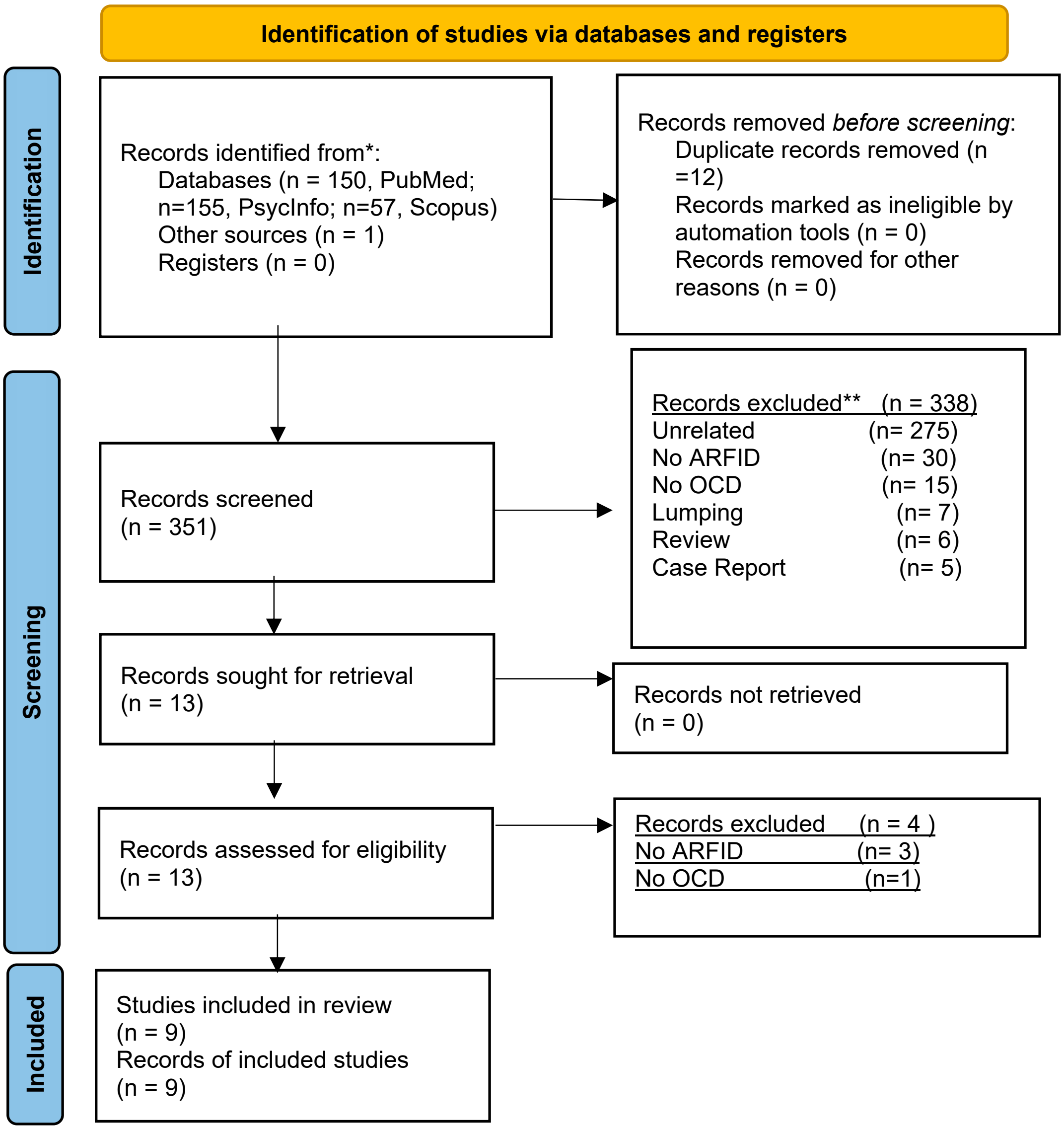

*Consider, if feasible to do so, reporting the number of records identified from each database or register searched (rather than the total number across all databases/registers).

**If automation tools were used, indicate how many records were excluded by a human and how many were excluded by automation tools. *No automation tools were used*

From: Page MJ, McKenzie JE, Bossuyt PM, Boutron I, Hoffmann TC, Mulrow CD, et al. The PRISMA 2020 statement: an updated guideline for reporting systematic reviews. BMJ 2021;372:n71. doi: 10.1136/bmj.n71

Supplementary Figure S2. PRISMA Checklist.

Section and Topic	Item #	Checklist item	Location where item is reported
**TITLE**			
Title	1	Identify the report as a systematic review.	Title
**ABSTRACT**			
Abstract	2	See the PRISMA 2020 for Abstracts checklist.	Abstract
**INTRODUCTION**			
Rationale	3	Describe the rationale for the review in the context of existing knowledge.	Introduction
Objectives	4	Provide an explicit statement of the objective(s) or question(s) the review addresses.	Introduction
**METHODS**			
Eligibility criteria	5	Specify the inclusion and exclusion criteria for the review and how studies were grouped for the syntheses.	Methods
Information sources	6	Specify all databases, registers, websites, organisations, reference lists and other sources searched or consulted to identify studies. Specify the date when each source was last searched or consulted.	Methods
Search strategy	7	Present the full search strategies for all databases, registers and websites, including any filters and limits used.	Methods
Selection process	8	Specify the methods used to decide whether a study met the inclusion criteria of the review, including how many reviewers screened each record and each report retrieved, whether they worked independently, and if applicable, details of automation tools used in the process.	Methods
Data collection process	9	Specify the methods used to collect data from reports, including how many reviewers collected data from each report, whether they worked independently, any processes for obtaining or confirming data from study investigators, and if applicable, details of automation tools used in the process.	Methods<br>Acknowledgements
Data items	10a	List and define all outcomes for which data were sought. Specify whether all results that were compatible with each outcome domain in each study were sought (e.g. for all measures, time points, analyses), and if not, the methods used to decide which results to collect.	Methods<br>Supplements
	10b	List and define all other variables for which data were sought (e.g. participant and intervention characteristics, funding sources). Describe any assumptions made about any missing or unclear information.	Supplements
Study risk of bias assessment	11	Specify the methods used to assess risk of bias in the included studies, including details of the tool(s) used, how many reviewers assessed each study and whether they worked independently, and if applicable, details of automation tools used in the process.	Methods<br>Supplements
Effect measures	12	Specify for each outcome the effect measure(s) (e.g. risk ratio, mean difference) used in the synthesis or presentation of results.	N/A
Synthesis methods	13a	Describe the processes used to decide which studies were eligible for each synthesis (e.g. tabulating the study intervention characteristics and comparing against the planned groups for each synthesis (item #5)).	Methods
	13b	Describe any methods required to prepare the data for presentation or synthesis, such as handling of missing summary statistics, or data conversions.	Methods
	13c	Describe any methods used to tabulate or visually display results of individual studies and syntheses.	Methods
	13d	Describe any methods used to synthesize results and provide a rationale for the choice(s). If meta-analysis was performed, describe the model(s), method(s) to identify the presence and extent of statistical heterogeneity, and software package(s) used.	Methods
	13e	Describe any methods used to explore possible causes of heterogeneity among study results (e.g. subgroup analysis, meta-regression).	N/A
	13f	Describe any sensitivity analyses conducted to assess robustness of the synthesized results.	N/A
Reporting bias assessment	14	Describe any methods used to assess risk of bias due to missing results in a synthesis (arising from reporting biases).	Methods<br>Supplements
Certainty assessment	15	Describe any methods used to assess certainty (or confidence) in the body of evidence for an outcome.	Methods
**RESULTS**			
Study selection	16a	Describe the results of the search and selection process, from the number of records identified in the search to the number of studies included in the review, ideally using a flow diagram.	Results<br>Supplements
	16b	Cite studies that might appear to meet the inclusion criteria, but which were excluded, and explain why they were excluded.	Supplements
Study characteristics	17	Cite each included study and present its characteristics.	Results
Risk of bias in studies	18	Present assessments of risk of bias for each included study.	Supplements<br>Limitations and strengths
Results of individual studies	19	For all outcomes, present, for each study: (a) summary statistics for each group (where appropriate) and (b) an effect estimate and its precision (e.g. confidence/credible interval), ideally using structured tables or plots.	Table 1<br>Results
Results of syntheses	20a	For each synthesis, briefly summarise the characteristics and risk of bias among contributing studies.	Results<br>Supplements
	20b	Present results of all statistical syntheses conducted. If meta-analysis was done, present for each the summary estimate and its precision (e.g. confidence/credible	N/A

Section and Topic	Item #	Checklist item	Location where item is reported
		interval) and measures of statistical heterogeneity. If comparing groups, describe the direction of the effect.	
	20c	Present results of all investigations of possible causes of heterogeneity among study results.	Discussion<br>Limitations and strengths
	20d	Present results of all sensitivity analyses conducted to assess the robustness of the synthesized results.	N/A
Reporting biases	21	Present assessments of risk of bias due to missing results (arising from reporting biases) for each synthesis assessed.	Supplements<br>Limitations and strengths
Certainty of evidence	22	Present assessments of certainty (or confidence) in the body of evidence for each outcome assessed.	Supplements<br>Results
**DISCUSSION**			
Discussion	23a	Provide a general interpretation of the results in the context of other evidence.	Discussion
	23b	Discuss any limitations of the evidence included in the review.	Limitations and strengths
	23c	Discuss any limitations of the review processes used.	Limitations and strengths
	23d	Discuss implications of the results for practice, policy, and future research.	Conclusions
**OTHER INFORMATION**			
Registration and protocol	24a	Provide registration information for the review, including register name and registration number, or state that the review was not registered.	N/A
	24b	Indicate where the review protocol can be accessed, or state that a protocol was not prepared.	N/A
	24c	Describe and explain any amendments to information provided at registration or in the protocol.	N/A
Support	25	Describe sources of financial or non-financial support for the review, and the role of the funders or sponsors in the review.	Funding
Competing interests	26	Declare any competing interests of review authors.	Disclosure statement
Availability of data, code and other materials	27	Report which of the following are publicly available and where they can be found: template data collection forms; data extracted from included studies; data used for all analyses; analytic code; any other materials used in the review.	Supplements

From: Page MJ, McKenzie JE, Bossuyt PM, Boutron I, Hoffmann TC, Mulrow CD, et al. The PRISMA 2020 statement: an updated guideline for reporting systematic reviews. BMJ 2021;372:n71. doi: 10.1136/bmj.n71

For more information, visit: http://www.prisma-statement.org/

Supplementary Table S1. Table of Included/excluded studies

	Search from PUBMED/MEDLINE	
1.	GeneReviews(®) [Internet]. Seattle (WA): University of Washington, Seattle; 1993–2024. 2017 Aug 10. TRIO-Related Neurodevelopmental Disorder. Varvagiannis K , Vissers LELM , Baralle D , de Vries BBA , Gazdagh G .	Unrelated
2.	Int J Eat Disord. 2020 Oct;53 :1636-1646. doi: 10.1002/eat.23355. Epub 2020 Aug 9. Cognitive-behavioral therapy for avoidant/restrictive food intake disorder: Feasibility, acceptability, and proof-of-concept for children and adolescents. Thomas JJ , Becker KR , Kuhnle MC , Jo JH , Harshman SG , Wons OB , Keshishian AC , Hauser K , Breithaupt L , Liebman RE , Misra M , Wilhelm S , Lawson EA , Eddy KT .	No OCD
3.	Child Psychiatry Hum Dev. 2016 Apr;47 :281-90. doi: 10.1007/s10578-015-0565-8. Obsessive-Compulsive Personality Traits in Youth with Obsessive-Compulsive Disorder. Park JM , Storch EA , Pinto A , Lewin AB .	No ARFID
4.	Transl Psychiatry. 2021 Feb 2;11 :91. doi: 10.1038/s41398-020-01121-9. Genome-wide association study of pediatric obsessive-compulsive traits: shared genetic risk between traits and disorder. Burton CL , Lemire M , Xiao B , Corfield EC , Erdman L , Bralten J , Poelmans G , Yu D , Shaheen SM , Goodale T , Sinopoli VM ; OCD Working Group of the Psychiatric Genomics	No ARFID

	Consortium; Soreni N , Hanna GL , Fitzgerald KD , Rosenberg D , Nestadt G , Paterson AD , Strug LJ , Schachar RJ , Crosbie J , Arnold PD .	
5.	Psychiatry Res Neuroimaging. 2019 Mar 30;285:40-46. doi: 10.1016/j.pscychresns.2019.01.012. Epub 2019 Jan 30. Corticostriatal-limbic correlates of sub-clinical obsessive-compulsive traits. Kubota Y , Sato W , Kochiyama T , Uono S , Yoshimura S , Sawada R , Toichi M .	No ARFID
6.	J Anxiety Disord. 2014 Mar;28 :148-53. doi: 10.1016/j.janxdis.2013.05.002. Epub 2013 May 27. Avoidance and behavioural flexibility in obsessive compulsive disorder. Hassoulas A , McHugh L , Reed P .	No ARFID
7.	J Psychol Psychother. 2013;7:3. Generalization of Conditioned Fear and Obsessive-Compulsive Traits. Kaczkurkin AN , Lissek S .	No ARFID
8.	J Exp Psychol Gen. 2021 Apr;150 :739-755. doi: 10.1037/xge0000966. Epub 2020 Oct 29. Anxious and obsessive-compulsive traits are independently associated with valuation of noninstrumental information. Bennett D , Sutcliffe K , Tan NP , Smillie LD , Bode S .	Unrelated
9.	Psychol Rep. 2008 Oct;103 :485-98. doi: 10.2466/pr0.103.2.485-498. A meta-analysis examining the relations among pathological gambling, obsessive-compulsive disorder, and obsessive-compulsive traits. Durdle H , Gorey KM, Stewart SH.	No ARFID
10.	medRxiv [Preprint]. 2024 Mar 15:2024.03.10.24304003. doi: 10.1101/2024.03.10.24304003. Co-existing mental and somatic conditions in Swedish children with the avoidant restrictive food intake disorder phenotype. Wronski ML , Kuja-Halkola R , Hedlund E , Martini MI , Lichtenstein P , Lundström S , Larsson H , Taylor MJ , Micali N , Bulik CM , Dinkler L .	Included
11.	Psychol Res Behav Manag. 2017 Mar 30;10:85-95. doi: 10.2147/PRBM.S113093. eCollection 2017. Compulsive exercise: links, risks and challenges faced. Lichtenstein MB, Hinze CJ , Emborg B , Thomsen F , Hemmingsen SD .	Unrelated
12.	J Psychiatr Res. 2022 Apr;148:21-26. doi: 10.1016/j.jpsychires.2022.01.029. Epub 2022 Jan 18. Individual obsessive-compulsive traits are associated with poorer adjustment to the easing of COVID-19 restrictions. Fineberg NA , Pellegrini L , Burkauskas J , Clarke A , Laws KR .	No ARFID
13.	J Am Acad Child Adolesc Psychiatry. 2016 Apr;55 :310-318.e4. doi: 10.1016/j.jaac.2016.01.008. Epub 2016 Feb 4. The Toronto Obsessive-Compulsive Scale: Psychometrics of a Dimensional Measure of Obsessive-Compulsive Traits. Park LS , Burton CL , Dupuis A , Shan J , Storch EA , Crosbie J , Schachar RJ , Arnold PD .	Unrelated
14.	Eur Child Adolesc Psychiatry. 2010 Jan;19 :17-24. doi: 10.1007/s00787-009-0035-6. Epub 2009 Jun 26.Obsessive-compulsive traits in children and adolescents with Asperger syndrome. Ruta L , Mugno D, D'Arrigo VG, Vitiello B, Mazzone L.	No ARFID
15.	J Eat Disord. 2016 Oct 29;4:26. doi: 10.1186/s40337-016-0110-6. eCollection 2016. Adult picky eaters with symptoms of avoidant/restrictive	Included

	food intake disorder: comparable distress and comorbidity but different eating behaviors compared to those with disordered eating symptoms. Zickgraf HF , Franklin ME , Rozin P .	
16.	Brain Sci. 2022 Nov 17;12 :1563. doi: 10.3390/brainsci12111563. Efficacy of Onabotulinum Toxin A on Obsessive-Compulsive Traits in a Population of Chronic Migraine Patients. Viticchi G , Falsetti L , Salvemini S , Bartolini M , Paolucci S , Buratti L , Silvestrini M .	Unrelated
17.	Iran J Psychiatry Behav Sci. 2015 Sep;9 :e264. doi: 10.17795/ijpbs-264. Epub 2015 Sep 23.Comparison of Perfectionism and Related Positive-Negative Dimension in People With High Traits on Obsessive Compulsive and Eating Disorder Characteristics. Yahghoubi H , Mohammadzadeh A .	No ARFID
18.	Tidsskr Nor Laegeforen. 2009 Apr 30;129 :877-81. doi: 10.4045/tidsskr.08.0509. [Self-harm and eating disorders].[Article in Norwegian] Skårderud F , Sommerfeldt B.	No OCD
19.	J Am Coll Health. 2022 Oct;70 :1975-1980. doi: 10.1080/07448481.2020.1841769. Epub 2020 Nov 12. Are the obsessive-compulsive traits a moderator for the relationship between autism and anorexia? A cross-sectional study among university students. Acikel SB , Cikili Y .	No ARFID
20.	Medicina (Kaunas). 2024 Jun 23;60 :1027. doi: 10.3390/medicina60071027. The Role of Obsessive Compulsive Traits in Fibromyalgia: Is Pain-Related Obsessive Ideation Involved in Pathogenesis? Lugassy-Galper BE , Amital M , Amital H , Buskila D , Amital D .	Unrelated
21.	Psych J. 2020 Oct;9 :749-759. doi: 10.1002/pchj.372. Epub 2020 Jul 16.Schizotypal and obsessive-compulsive traits: Co-occurrence rate and relationship with executive function, emotion experience, and emotion expressivity in college students. Shan HD , Zhang RT , Jiang SY , Wang YM , Liu YF , Cheung EFC , Chan RCK .	Unrelated
22.	Psychol Res Behav Manag. 2014 Mar 10;7:93-101. doi: 10.2147/PRBM.S43666. eCollection 2014. Influence of religious aspects and personal beliefs on psychological behavior: focus on anxiety disorders. Agorastos A , Demiralay C , Huber CG .	Unrelated
23.	Psychiatry Res Neuroimaging. 2020 Nov 30;305:111170. doi: 10.1016/j.pscychresns.2020.111170. Epub 2020 Aug 18. Altered default mode network functional connectivity in individuals with co-occurrence of schizotypy and obsessive-compulsive traits. Wang YM , Cai XL , Zhou HY , Zhang RT , Zhang YJ , Wang YY , Cheung EFC , Chan RCK .	Unrelated
24.	J Neurol Neurosurg Psychiatry. 2001 Mar;70 :394-6. doi: 10.1136/jnnp.70.3.394. Obsessive-compulsive symptoms in Parkinson's disease. Alegret M , Junqué C, Valldeoriola F, Vendrell P, Martí MJ, Tolosa E.	Unrelated
25.	Autism. 2007 Mar;11 :101-10. doi: 10.1177/1362361307075699. An autistic dimension: a proposed subtype of obsessive-compulsive disorder. Bejerot S .	No ARFID

26.	Percept Mot Skills. 2022 Jun;129 :399-414. doi: 10.1177/00315125221086254. Epub 2022 Apr 19. How Untidiness Moves the Motor System. Fiori F , Ciricugno A , Rusconi ML , Slaby RJ , Cattaneo Z .	Unrelated
27.	Asian J Psychiatr. 2010 Dec;3 :194-9. doi: 10.1016/j.ajp.2010.09.005. Epub 2010 Nov 20. Body dysmorphic traits and personality disorder patterns in rhinoplasty seekers. Barahmand U , Mozdsetan N, Narimani M.	Unrelated
28.	Eur Eat Disord Rev. 2014 Jul;22 :237-42. doi: 10.1002/erv.2295. Epub 2014 May 23. Childhood obsessive-compulsive traits in anorexia nervosa patients, their unaffected sisters and healthy controls: a retrospective study. Degortes D , Zanetti T, Tenconi E, Santonastaso P, Favaro A.	No ARFID
29.	J Autism Dev Disord. 2023 Feb 4. doi: 10.1007/s10803-022-05886-4. Online ahead of print. Brief Report: Local-Global Processing and Co-occurrence of Anxiety, Autistic and Obsessive-Compulsive Traits in a Non-clinical Sample. Retzler C , Retzler J .	Unrelated
30.	Front Pediatr. 2023 Sep 21;11:1170379. doi: 10.3389/fped.2023.1170379. eCollection 2023. Estimate of the incidence of PANDAS and PANS in 3 primary care populations. Wald ER , Eickhoff J , Flood GE , Heinz MV , Liu D , Agrawal A , Morse RP , Raney VM , Veerapandiyan A , Madan JC .	Unrelated
31.	Depress Anxiety. 2020 Aug;37 :760-770. doi: 10.1002/da.22996. Epub 2020 Feb 24. Serotonin system genes and hoarding with and without other obsessive-compulsive traits in a population-based, pediatric sample: A genetic association study. Sinopoli VM , Erdman L , Burton CL , Park LS , Dupuis A , Shan J , Goodale T , Shaheen SM , Crosbie J , Schachar RJ , Arnold PD .	Unrelated
32.	Cureus. 2022 Mar 7;14 :e22939. doi: 10.7759/cureus.22939. eCollection 2022 Mar. Personality Traits, Dimensions, and Suicidal Behavior in Posttraumatic Stress Disorder: Results From a Cross-Sectional Study in a Mexican Hospital. Medina JC , Merlín García I , Aguilar Salas I .	Unrelated
33.	Mol Autism. 2017 Oct 24;8:57. doi: 10.1186/s13229-017-0172-6. eCollection 2017. Prospective investigation of FOXP1 syndrome. Siper PM, De Rubeis S, Trelles MDP , Durkin A , Di Marino D , Muratet F , Frank Y , Lozano R , Eichler EE , Kelly M , Beighley J , Gerdts J , Wallace AS , Mefford HC , Bernier RA , Kolevzon A(#) , Buxbaum JD(#) .	Unrelated
34.	Int J Eat Disord. 2024 Oct;57 :1999-2005. doi: 10.1002/eat.24218. Epub 2024 Apr 17. Lifetime and current mental health based on avoidant/restrictive food intake disorder history versus other eating disorder history in the Healthy Minds Study. Richson BN , Zickgraf HF .	Included
35.	J Manipulative Physiol Ther. 2020 Nov-Dec;43 :891-900. doi: 10.1016/j.jmpt.2019.10.014. Epub 2020 Sep 4. Chest Wall Mobility: Identification of Underlying Predictors. Mustafaoğlu R , Birinci T , Mutlu EK , Ozdincler AR .	Unrelated
36.	Int J Psychiatry Clin Pract. 2021 Jun;25 :152-163. doi: 10.1080/13651501.2021.1879159. Epub 2021 Feb 15. COVID-19 pandemic	Unrelated

	and mental health in Lebanon: a cross-sectional study. El Othman R , Touma E , El Othman R , Haddad C , Hallit R , Obeid S , Salameh P , Hallit S .	
37.	J Can Acad Child Adolesc Psychiatry. 2017 Winter;26 :59-61. Epub 2017 Mar 1. Fear of Vomiting and Low Body Weight in Two Pediatric Patients: Diagnostic Challenges. Maertens C , Couturier J , Grant C , Johnson N .	Unrelated
38.	Clin Otolaryngol. 2009 Oct;34 :423-30. doi: 10.1111/j.1749-4486.2009.02013.x. Is musical hallucination an otological phenomenon? a review of the literature. Cope TE , Baguley DM.	Review
39.	Perspect Psychiatr Care. 2021 Apr;57 :717-725. doi: 10.1111/ppc.12603. Epub 2020 Aug 10. Quality of life and infertility stress in homologous and heterologous medically assisted reproduction: The role of common and specific psychopathological traits. Pozza A , Dèttore D , Coccia ME .	Unrelated
40.	Tremor Other Hyperkinet Mov (N Y). 2021 Jul 8;11:27. doi: 10.5334/tohm.636. eCollection 2021. The Dutch Yips Study: Results of a Survey Among Golfers. van Wensen E , van der Zaag-Loonen HJ , van de Warrenburg BP .	Unrelated
41.	Neurodegener Dis. 2019;19(5-6):171-177. doi: 10.1159/000507447. Epub 2020 May 26. Neuropsychiatric Traits Associated with Refractory Impulse Control Disorder in Parkinson's Disease. Choi JH , Lee JY , Jeon B , Koh SB , Yoon WT , Lee HW , Kwon OD , Kim JW , Kim JM , Ma HI , Kim HT , Baik JS , Cho JW .	Unrelated
42.	Egypt J Neurol Psychiatr Neurosurg. 2022;58:102. doi: 10.1186/s41983-022-00538-x. Epub 2022 Sep 3. Behavioral and psychological symptoms in neurodegenerative dementias: harbinger, follower, or constant collateral? Dubey S , Dubey MJ , Ghosh R , Mukherjee D , Pandit A , Benito-León J .	Unrelated
43.	J Autism Dev Disord. 2024 Apr 12. doi: 10.1007/s10803-024-06336-z. Online ahead of print. Treating Obsessive Compulsive Disorder in Adolescents and Adults with Down Syndrome: Results from a Scoping Rapid Review. Fodstad JC , Russell R , Bullington M , Jones LB , Iticovici M , Meudt E .	Review
44.	Eat Weight Disord. 2022 Dec;27 :3695-3711. doi: 10.1007/s40519-022-01512-5. Epub 2022 Nov 27. A consensus document on definition and diagnostic criteria for orthorexia nervosa. Donini LM , Barrada JR, Barthels F, Dunn TM, Babeau C , Brytek-Matera A , Cena H , Cerolini S , Cho HH , Coimbra M , Cuzzolaro M , Ferreira C , Galfano V, Grammatikopoulou MG , Hallit S , Håman L , Hay P , Jimbo M , Lasson C , Lindgren EC , McGregor R , Minnetti M , Mocini E , Obeid S , Oberle CD , Onieva-Zafra MD , Opitz MC , Parra-Fernández ML , Pietrowsky R , Plasonja N , Poggiogalle E , Rigó A, Rodgers RF, Roncero M, Saldaña C, Segura-Garcia C, Setnick J, Shin JY , Spitoni G , Strahler J, Stroebele-Benschop N, Todisco P, Vacca M , Valente M, Varga M, Zagaria A , Zickgraf HF, Reynolds RC, Lombardo C.	Unrelated
45.	Crim Behav Ment Health. 2015 Feb;25 :66-78. doi: 10.1002/cbm.1925. Epub 2014 Aug 11. Single-victim and serial sexual homicide offenders: differences in crime, paraphilias and personality traits. Chan HC , Beauregard E, Myers WC.	Unrelated

46.	Eur Eat Disord Rev. 2024 Nov 28. doi: 10.1002/erv.3160. Online ahead of print. Anxiety, Obsessive-Compulsive, and Depressive Symptom Presentation and Change Throughout Routine Eating Disorder Treatment. Velimirović M , Robison M , Abber S , Duffy A , Rienecke RD , Manwaring J , Blalock DV , Riddle M , Mehler PS , Joiner TE .	Included
47.	Pharmaceuticals (Basel). 2009 Nov 16;2 :82-93. doi: 10.3390/ph2030082. Obsessive-Compulsive and Post Traumatic Avoidance Symptoms Influence the Response to Antihypertensive Therapy: Relevance in Uncontrolled Hypertension. Realdi A , Favaro A , Santonastaso P , Nuti M , Parotto E , Inverso G , Leoni M , Macchini L , Vettore F , Calo L, Semplicini A .	Unrelated
48.	Alcohol Clin Exp Res. 2002 Aug;26 :1223-7. doi: 10.1097/01.ALC.0000023985.20126.DC. Co-occurrence of obsessive-compulsive personality traits in young and middle-aged Japanese alcohol-dependent men. Suzuki K , Muramatsu T, Takeda A, Shirakura K.	Unrelated
49.	Appetite. 2019 Mar 1;134:40-49. doi: 10.1016/j.appet.2018.12.006. Epub 2018 Dec 10. Disentangling orthorexia nervosa from healthy eating and other eating disorder symptoms: Relationships with clinical impairment, comorbidity, and self-reported food choices. Zickgraf HF , Ellis JM , Essayli JH .	Unrelated
50.	Mol Genet Metab. 2024 May;142 :108363. doi: 10.1016/j.ymgme.2024.108363. Epub 2024 Mar 4. Consensus guidelines for the diagnosis and management of succinic semialdehyde dehydrogenase deficiency. Tokatly Latzer I , Bertoldi M , Blau N , DiBacco ML , Elsea SH , García-Cazorla À , Gibson KM , Gropman AL , Hanson E , Hoffman C , Jeltsch K , Juliá-Palacios N , Knerr I , Lee HHC , Malaspina P , McConnell A , Opladen T , Oppebøen M , Rotenberg A , Walterfang M , Wang-Tso L , Wevers RA , Roullet JB , Pearl PL .	Unrelated
51.	Appetite. 2015 Jul;90:219-28. doi: 10.1016/j.appet.2015.03.001. Epub 2015 Mar 5. Adult picky eating. Phenomenology, taste sensitivity, and psychological correlates. Kauer J , Pelchat ML , Rozin P , Zickgraf HF .	No ARFID
52.	JCPP Adv. 2021 Dec 3;1 :e12056. doi: 10.1002/jcv2.12056. eCollection 2021 Dec. Clinical validation of the parent-report Toronto Obsessive-Compulsive Scale (TOCS): A pediatric open-source rating scale. Lambe LJ , Burton CL , Anagnostou E , Kelley E , Nicolson R , Georgiades S , Soreni N , Schachar RJ , Hanna GL , Arnold PD , Crosbie J .	No ARFID
53.	Asian J Psychiatr. 2019 Aug;44:209-216. doi: 10.1016/j.ajp.2019.08.005. Epub 2019 Aug 5. Network analysis of schizotypal personality traits and their association with other subclinical psychiatric features. Zhang RT , Zhou HY , Wang YM , Yang ZY , Wang Y , So SH , Chiu CD , Leung PWL , Cheung EFC , Chan RCK .	Unrelated
54.	Braz J Psychiatry. 2023 Mar 11;45 :11-19. doi: 10.47626/1516-4446-2022-2650. Heterozygosity for neuronal ceroid lipofuscinosis predisposes to bipolar disorder. Privitera F , Trusso MA , Valentino F , Doddato G , Fallerini C , Brunelli G , D'Aurizio R , Furini S , Goracci A , Fagiolini A , Mari F , Renieri A , Ariani F .	Unrelated

55.	Epilepsy Behav. 2020 Apr;105:106958. doi: 10.1016/j.yebeh.2020.106958. Epub 2020 Feb 22. Dysfunctional personality beliefs and executive performance in patients with juvenile myoclonic epilepsy. Taura M , Gama AP , Sousa AVM , Noffs MHS , Alonso NB , Yacubian EM , Guilhoto LM .	Unrelated
56.	J Nerv Ment Dis. 2014 Apr;202 :319-23. doi: 10.1097/NMD.0000000000000125. Maladaptive personality traits increase subjectively during the course of schizophrenia spectrum disorders. Schroeder K , Naber D, Huber CG.	Unrelated
57.	Ann Clin Psychiatry. 1995 Jun;7 :65-70. doi: 10.3109/10401239509149029. Complaints of constipation in obsessive-compulsive disorder. North CS , Napier M, Alpers DH, Spitznagel EL.	No ARFID
58.	Eat Weight Disord. 2019 Feb;24 :13-20. doi: 10.1007/s40519-018-0592-0. Epub 2018 Oct 20. An unhealthy health behavior: analysis of orthorexic tendencies among Hungarian gym attendees. Bóna E , Szél Z , Kiss D , Gyarmathy VA .	Unrelated
59.	Nervenarzt. 1999 Jan;70 :1-10. doi: 10.1007/s001150050394. [Relationship between tics and compulsion]. [Article in German] Moll GH , Rothenberger A.	No ARFID
60.	Noro Psikiyatr Ars. 2024 Aug 9;67 :248-254. doi: 10.29399/npa.28630. eCollection 2024. Sex-Specific Correlations Between Misophonia Symptoms and ADHD, OCD, and Autism-Related Traits in Adolescent Outpatients. Herdi O , Yıldırım F .	Unrelated
61.	Am J Med Genet B Neuropsychiatr Genet. 2012 Jun;159B :376-82. doi: 10.1002/ajmg.b.32040. Epub 2012 Mar 20. Evidence for a genetic overlap between body dysmorphic concerns and obsessive-compulsive symptoms in an adult female community twin sample. Monzani B , Rijsdijk F, Iervolino AC, Anson M, Cherkas L, Mataix-Cols D.	Unrelated
62.	Urology. 2014 Sep;84 :685-8. doi: 10.1016/j.urology.2014.04.046. Phantom urinary incontinence in children with bladder-bowel dysfunction. Arlen AM , Dewhurst LL , Kirsch SS , Dingle AD , Scherz HC , Kirsch AJ .	Unrelated
63.	J Intellect Disabil Res. 2005 Jun;49(Pt 6):449-56. doi: 10.1111/j.1365-2788.2005.00690.x. ADHD symptoms and insistence on sameness in Prader-Willi syndrome. Wigren M , Hansen S.	Unrelated
64.	J Child Adolesc Psychopharmacol. 2015 Feb;25 :48-56. doi: 10.1089/cap.2014.0063. Epub 2014 Oct 20. Disordered eating and food restrictions in children with PANDAS/PANS. Toufexis MD , Hommer R, Gerardi DM, Grant P, Rothschild L, D'Souza P, Williams K, Leckman J, Swedo SE, Murphy TK.	Unrelated
65.	Br J Pharmacol. 2017 Oct;174 :3333-3345. doi: 10.1111/bph.13952. Epub 2017 Aug 19. Methoxetamine affects brain processing involved in emotional response in rats. Zanda MT , Fadda P , Antinori S , Di Chio M , Fratta W , Chiamulera C , Fattore L .	Unrelated
66.	Neurosci Biobehav Rev. 2019 Aug;103:133-149. doi: 10.1016/j.neubiorev.2019.06.013. Epub 2019 Jun 18. ERN as a	Unrelated

	transdiagnostic marker of the internalizing-externalizing spectrum: A dissociable meta-analytic effect. Pasion R , Barbosa F .	
67.	Assessment. 2018 Jul;25 :578-588. doi: 10.1177/1073191116659740. Epub 2016 Jul 22. Comparing Two Short Forms of the Hewitt-Flett Multidimensional Perfectionism Scale. Stoeber J .	Unrelated
68.	Front Psychol. 2022 Dec 21;13:902807. doi: 10.3389/fpsyg.2022.902807. eCollection 2022. A nomological network for misophonia in two German samples using the S-Five model for misophonia. Remmert N , Jebens A , Gruzman R , Gregory J , Vitoratou S .	Unrelated
69.	Vertex. 2004 Sep-Nov;15(57):175-9. [Eating behaviors, attitudes and obsessive compulsive traits in adolescents of Buenos Aires City]. [Article in Spanish] Leiderman EA , Triskier FJ.	No ARFID
70.	Psych J. 2020 Apr;9 :223-233. doi: 10.1002/pchj.331. Epub 2019 Dec 17. Network structure of anticipatory pleasure and risk features: Evidence from a large college sample. Zhang RT , Wang Y , Yang ZY , Li Y , Wang YM , Cheung EFC , Shum DHK , Yang TX , Barkus EJ , Chan RCK .	Unrelated
71.	J Eat Disord. 2018 Feb 5;6:2.doi: 10.1186/s40337-018-0188-0.eCollection 2018.Relationships between compulsive exercise, quality of life, psychological distress and motivation to change in adults with anorexia nervosa.Young S,Touyz S,Meyer C,Arcelus J,Rhodes P,Madden S, Pike K,Attia E,Crosby RD,Hay P.	Unrelated
72.	PLoS One. 2023 May 19;18 :e0277446.doi: 10.1371/journal.pone.0277446.eCollection 2023. Mice lacking Ptprd exhibit deficits in goal-directed behavior and female-specific impairments in sensorimotor gating.Ho EV , Welch A , Thompson SL , Knowles JA , Dulawa SC.	Unrelated
73.	Ann Clin Psychiatry. 2018 May;30:102-112.Age at first sexual activity: Clinical and cognitive associations.Harries MD , Paglia HA, Redden SA, Grant JE.	Unrelated
74.	J Eat Disord. 2013 Apr 15;1:13. doi: 10.1186/2050-2974-1-13.eCollection 2013.The cognitive-interpersonal maintenance model of anorexia nervosa revisited: a summary of the evidence for cognitive, socio-emotional and interpersonal predisposing and perpetuating factors.Treasure J , Schmidt U .	Unrelated
75.	Appetite. 2019 Sep 1;140:50-75.doi:10.1016/j.appet.2019.05.005.Epub 2019 May 7.Orthorexia nervosa: A review of psychosocial risk factors.McComb SE,Mills JS.	Review
76.	J Psychiatr Res.2004 Sep-Oct;38:545-52.doi:10.1016/j.jpsychires.2004.03.00.Set shifting inanorexia nervosa:an examination before and after weight gain,in full recovery and relationship to childhood and adult OCPD traits.Tchanturia K , Morris RG, Anderluh MB, Collier DA, Nikolaou V, Treasure J.	Unrelated

77.	Behav Neurol.1992;5 :27-32.doi:10.3233/BEN-1992-5105.Do some cases of anorexia nervosa reflect underlying autistic-like conditions? Gillberg C , Råstam M .	Unrelated
78.	PLoS One.2011;6 :e20835.doi:10.1371/journal.pone.0020835.Epub 2011 Jun 13.A behavioral comparison of male and female adults with high functioning autism spectrum conditions.Lai MC,Lombardo MV,Pasco G,Ruigrok AN,Wheelwright SJ,Sadek SA,Chakrabarti B; MRC AIMS Consortium; Baron-Cohen S.	Unrelated
79.	Arch Neurol.2003 Nov;60:1595-8.doi:10.1001/archneur.60.11.1595.Migraine headache in patients with Tourette syndrome.Kwak C , Vuong KD, Jankovic J.	Unrelated
80.	J Med Assoc Thai. 2015 Mar;98 Suppl 2:S28-37.Mental health and quality of life among Thai psychiatrists.Kosulwit L.	Unrelated
81.	Brain Sci. 2022 Oct 26;12 :1448.doi:10.3390/brainsci12111448.Cognition,Behavior,Sexuality, and Autonomic Responses of Women with Hypothalamic Amenorrhea.Pruneti C,Guidotti S.	Unrelated
82.	J Neurol.2017 Jan;264 :40-48.doi:10.1007/s00415-016-8314-x Epub 2016 Oct 19.Impulse control behaviors and subthalamic deep brain stimulation in Parkinson disease.Merola A, Romagnolo A,Rizzi L,Rizzone MG,Zibetti M,Lanotte M,Mandybur G,Duker AP,Espay AJ,Lopiano L.	Unrelated
83.	J Med Genet.2016 May;53:318-29.doi:10.1136/jmedgenet-2015103416.Epub2016Jan13.Cystic cerebellar dysplasia and biallelic LAMA1 mutations:a lamininopathy associated with tics,obsessive compulsive traits and myopia due to cell adhesion and migration defects. Vilboux T,Malicdan MC,Chang YM,Guo J,Zerfas PM, Bryan MM,VemulapalliM,Mullikin JC,Kirby M,Anderson SM,Toro C,Gahl WA,Gunay-Aygun M.	Unrelated
84.	Cureus. 2024 Mar 14; 16:e56165. doi:10.7759/cureus.56165. eCollection 2024 Mar. Does Western or Chinese Zodiac Sign Predict COVID Infections and Death? Frugoli A, Parekh S, Diaz G.	Unrelated
85.	doi:10.3389/fnbeh.2023.1257417. eCollection 2023. Communal nesting differentially attenuates the impact of pre-weaning social isolation on behavior in male and female rats during adolescence and adulthood. Bratzu J, Ciscato M, Pisanu A, Talani G, Frau R, Porcu P, Diana M, Fumagalli F, Romualdi P, Rullo L, Trezza V, Ciccocioppo R, Sanna F, Fattore L.	Unrelated
86.	Clin Genet. 2011 Dec;80:523-31. doi:10.1111/j.1399-0004.2011.01688.x. Epub 2011 May 25. Hippocampal dysgenesis and variable neuropsychiatric phenotypes in patients with Bardet-Biedl syndrome underline complex CNS impact of primary cilia. Bennouna-Greene V, Kremer S, Stoetzel C, Christmann D, Schuster C, Durand M, Verloes A, Sigaudy S, Holder-Espinasse M, Godet J, Brandt C, Marion V, Danion A, Dietemann JL, Dollfus H.	Unrelated
87.	Eat Disord. 2024 Mar-Apr;32:195-211. doi:10.1080/10640266.2023.2293502. Epub 2023 Dec 14. Eating	Unrelated

	behaviours and personality characteristics of clinicians and researchers working in eating disorders. Poiani-Cordella C, Toh WL, Phillipou A.	
88.	Eat Behav. 2023 Aug;50:101751. doi:10.1016/j.eatbeh.2023.101751. Epub 2023 May 20. Examining the momentary relationships between body checking and eating disorder symptoms in women with anorexia nervosa. Goeden AV, Schaefer LM, Crosby RD, Peterson CB, Engel SG, Le Grange D, Crow SJ, Wonderlich SA.	Unrelated
89.	Int J Environ Res Public Health. 2020 Nov 18;17 :8550. doi:10.3390/ijerph17228550.Same Involvement, Different Reasons: How Personality Factors and Organizations Contribute to Heavy Work Investment.Mazzetti G , Guglielmi D , Schaufeli WB .	Unrelated
90.	Mol Autism. 2014 Dec 20;5 :56.doi: 10.1186/2040-2392-5-56.eCollection 2014.An examination of autism spectrum traits in adolescents with anorexia nervosa and their parents.Rhind C , Bonfioli E , Hibbs R , Goddard E , Macdonald P , Gowers S , Schmidt U , Tchanturia K ,Micali N(#) , Treasure J(#) .	Unrelated
91.	Sleep Med. 2010 Feb;11:218-20. doi:10.1016/j.sleep.2009.05.016. Epub 2010 Jan 12. Increased prevalence of nocturnal smoking in restless legs syndrome (RLS). Provini F, Antelmi E, Vignatelli L, Zaniboni A, Naldi G, Calandra-Buonaura G, Vetrugno R, Plazzi G, Pizza F, Montagna P.	Unrelated
92.	J Affect Disord. 2012 Dec 10;141(2-3):464-8. doi:10.1016/j.jad.2012.03.017. Epub 2012 Apr 3. Mood-congruent and mood-incongruent psychotic symptoms in major depression: the role of severity and personality. Tonna M, De Panfilis C, Marchesi C.	Unrelated
93.	Acta Neurol Scand. 2013 Jul;128:54-60. doi:10.1111/ane.12078. Epub 2013 Feb 13. Psychological functioning measures in patients with primary insomnia and sleep state misperception. Dittoni S, Mazza M, Losurdo A, Testani E, Di Giacopo R, Marano G, Di Nicola M, Farina B, Mariotti P, Mazza S, Della Marca G.	Unrelated
94.	J Clin Psychol. 2023 Oct;79:2364-2387. doi:10.1002/jclp.23552. Epub 2023 Jun 21. A symptom network model of misophonia: From heightened sensory sensitivity to clinical comorbidity. Andermane N, Bauer M, Simner J, Ward J.	Unrelated
95.	Psychiatry Res.2003 Jan 25;117 :11-6.doi:10.1016/s0165-1781(02)00304-9.Obsessive-compulsive behaviors in parents of multiplex autism families.Hollander E , King A, Delaney K, Smith CJ, Silverman JM.	No ARFID
96.	Int J Eat Disord. 1996 Mar;19 :147-57.doi:10.1002/(SICI)1098-108X(199603)19:2<147::AID-EAT5>3.0.CO;2-N.Familial eating concerns and psychopathological traits: causal implications of transgenerational effects.Steiger H , Stotland S, Trottier J, Ghadirian AM.	Unrelated
97.	Psychiatr Clin North Am.1994 Dec;17 :773-84.Characterologic subtypes of the borderline personality disorder. With a note on prognostic factors.Stone MH .	Unrelated

98.	Biosci Trends. 2010 Feb;4:4-8. A study of the relationship between mental health and menstrual abnormalities in female middle school students from postearthquake Wenchuan. Liu X, Yang Y, Yuan P, Zhang X, Han Y, Cao Y, Xiong G.	Unrelated
99.	J Pers Assess. 1995 Feb;64:132-44. doi:10.1207/s15327752jpa6401_9. Defense mechanisms and personality disorders: an empirical test of Millon's theory. Berman SM, McCann JT.	Unrelated
100.	J Neurol Neurosurg Psychiatry. 2019 Jan;90:30-37. doi:10.1136/jnnp-2018-318942. Epub 2018 Oct 25. Behavioural and trait changes in parkinsonian patients with impulse control disorder after switching from dopamine agonist to levodopa therapy: results of REIN-PD trial. Lee JY, Jeon B, Koh SB, Yoon WT, Lee HW, Kwon OD, Kim JW, Kim JM, Ma HI, Kim HT, Baik JS, Cho J.	Unrelated
101.	Psychiatry Res. 2011 May 30;187:401-8. doi:10.1016/j.psychres.2010.10.028. Epub 2010 Nov 20. Personality development characteristics of women with anorexia nervosa, their healthy siblings and healthy controls: What prevents and what relates to psychopathology? Amianto F, Abbate-Daga G, Morando S, Sobrero C, Fassino S.	Unrelated
102.	Intern Emerg Med. 2008 Dec;3:339-43. doi:10.1007/s11739-008-0166-7. Epub 2008 Jun 13. Psychosocial aspects and psychiatric disorders in young adults with thalassemia major. Messina G, Colombo E, Cassinerio E, Ferri F, Curti R, Altamura C, Cappellini MD.	Unrelated
103.	Int J Dev Disabil. 2022 Aug 24;70:518-529. doi:10.1080/20473869.2022.2113321. eCollection 2024. Characteristics of children with autism and unspecified intellectual developmental disorder (intellectual disability) presenting with severe self-injurious behaviours. Fong A, Friedlander R, Richardson A, Allen K, Zhang Q.	Unrelated
104.	Psychol Med. 2009 Jan;39:105-14. doi:10.1017/S0033291708003292. Epub 2008 Apr 1. Lifetime course of eating disorders: design and validity testing of a new strategy to define the eating disorders phenotype. Anderluh M, Tchanturia K, Rabe-Hesketh S, Collier D, Treasure J.	Unrelated
105.	J Clin Exp Neuropsychol. 2009 May;31:455-61. doi:10.1080/13803390802251378. An examination of decision making in bulimia nervosa. Liao PC, Uher R, Lawrence N, Treasure J, Schmidt U, Campbell IC, Collier DA, Tchanturia K.	Unrelated
106.	Nihon Rinsho.2007 Mar;65 :432-6.[Neurological background of Asperger's disorder and other pervasive developmental disorders].[Article in Japanese].Toichi M .	Unrelated
107.	Int J Eat Disord. 2002 Jul;32:72-8. doi:10.1002/eat.10072. Medical and psychiatric morbidity in obese women with and without binge eating. Bulik CM, Sullivan PF, Kendler KS.	Unrelated
108.	J Eat Disord. 2024 Jun 24;12:86. doi:10.1186/s40337-024-01048-2. Maladaptive exercise in eating disorders: lifetime and current impact on	Unrelated

	mental health and treatment seeking. Liao Z, Birgegård A, Monell E, Borg S, Bulik CM, Mantilla EF.	
109.	Int J Eat Disord. 2018 Jan;51:39-45. doi:10.1002/eat.22805. Epub 2017 Dec 7. Atypical anorexia nervosa is not related to brain structural changes in newly diagnosed adolescent patients. Olivo G, Solstrand Dahlberg L, Wiemerslage L, Swenne I, Zhukovsky C, Salonen-Ros H, Larsson EM, Gaudio S, Brooks SJ, Schiöth HB.	Unrelated
110.	Front Psychol. 2022 Apr 6;13:808379. doi:10.3389/fpsyg.2022.808379. eCollection 2022. Poorer Well-Being in Children With Misophonia: Evidence From the Sussex Misophonia Scale for Adolescents. Rinaldi LJ, Smees R, Ward J, Simner J.	Unrelated
111.	Alpha Psychiatry. 2024 Jan 1;25:23-29. doi:10.5152/alphapsychiatry.2024.231216. eCollection 2024 Jan. The Correlation Between Peripheral Blood Micro-Ribonucleic Acid Expression Level and Personality Disorder in Patients with Schizophrenia. Wei H, Kong L, Zhu X, Chen S, Zhang L, Niu W.	Unrelated
112.	Int J Eat Disord. 1994 Dec;16:381-93. doi:10.1002/1098-108x(199412)16:4<381::aid-eat2260160407>3.0.co;2-q. Convergent validity of the eating disorder inventory and the anorexia nervosa inventory for self-rating in an Austrian nonclinical population. Rathner G, Rumpold G.	Unrelated
113.	Plast Reconstr Surg. 2005 Sep 15;116:993-1002; discussion 1003-5. doi: 10.1097/01.prs.0000178395.19992.ca. Quality of life and affective distress in women seeking immediate versus delayed breast reconstruction after mastectomy for breast cancer. Roth RS, Lowery JC, Davis J, Wilkins EG.	Unrelated
114.	J Eat Disord. 2015 Apr 2;3:14. doi:10.1186/s40337-015-0050-6. eCollection 2015. Comparison in decision-making between bulimia nervosa, anorexia nervosa, and healthy women: influence of mood status and pathological eating concerns. Matsumoto J, Hirano Y, Numata N, Matzuzawa D, Murano S, Yokote K, Iyo M, Shimizu E, Nakazato M.	Unrelated
115.	Ital J Pediatr. 2019 Jun 26;45:74. doi:10.1186/s13052-019-0667-1. Eye movement desensitisation and reprocessing (EMDR) treatment associated with parent management training (PMT) for the acute symptoms in a patient with PANDAS syndrome: a case report. Guido CA, Zicari AM, Duse M, Spalice A.	Case Report
116.	J Neurol. 2008 Oct;255:1515-20. doi:10.1007/s00415-008-0955-y. Epub 2008 Sep 3. Neuropsychiatric symptoms three years after subthalamic DBS in PD patients: a case-control study. Castelli L, Zibetti M, Rizzi L, Caglio M, Lanotte M, Lopiano L.	Unrelated
117.	Rev Neurol. 2004 Nov 1-15;39:810-5. [Sydenham's chorea. A clinical analysis of 55 patients with a prolonged follow-up]. [Article in Spanish]. Díaz-Grez F, Lay-Son L, del Barrio Guerrero E, Vidal-González P.	Unrelated
118.	J Nerv Ment Dis. 1976 Oct;163:233-45. doi:10.1097/00005053-197610000-00002. MDA assisted psychotherapy with neurotic outpatients:	Unrelated

	a pilot study. Yensen R, Di Leo FB, Rhead JC, Richards WA, Soskin RA, Turek B, Kurland AA.	
119.	Arch Neurol. 2001 Aug;58:1223-7. doi:10.1001/archneur.58.8.1223. Effects of bilateral subthalamic stimulation on cognitive function in Parkinson disease. Alegret M, Junqué C, Valldeoriola F, Vendrell P, Pilleri M, Rumià J, Tolosa E.	Unrelated
120.	Front Psychol. 2024 Oct 22;15:1490147. doi:10.3389/fpsyg.2024.1490147. eCollection 2024. Proactive control for conflict resolution is intact in subclinical obsessive-compulsive individuals. Fornaro S, Visalli A, Viviani G, Ambrosini E, Vallesi A.	Unrelated
121.	J Psychoactive Drugs. 2009 Jun;41:135-43. doi:10.1080/02791072.2009.10399906. Nitrite inhalant abuse in antisocial youth: prevalence, patterns, and predictors. Hall MT, Howard MO.	Unrelated
122.	Genes Brain Behav. 2010 Aug;9:621-7. doi:10.1111/j.1601-183X.2010.00594.x. Epub 2010 May 18. Association of Neuregulin 1 rs3924999 genotype with antisaccades and smooth pursuit eye movements. Schmechtig A, Vassos E, Kumari V, Hutton SB, Collier DA, Morris RG, Williams SC, Ettinger U.	Unrelated
123.	Rev Neurol. 2003 May 1-15;36:837-40. [Nasu Hakola disease: a report of the first two cases in Bolivia]. [Article in Spanish]. Molina-Monasterios MC, Molina-Abecia H	Unrelated
124.	Cancer. 1999 May 15;85:2273-7. Effects of physical activity on the fatigue and psychologic status of cancer patients during chemotherapy. Dimeo FC, Stieglitz RD, Novelli-Fischer U, Fetscher S, Keul J.	Unrelated
125.	Brain Inj. 1999 Feb;13:125-30. doi:10.1080/026990599121782. The usefulness of the Brief Symptom Inventory in the neuropsychological evaluation of traumatic brain injury. Slaughter J, Johnstone G, Petroski G, Flax J.	Unrelated
126.	Eur Arch Psychiatry Clin Neurosci. 1998;248:171-9. doi:10.1007/s004060050035. The factor structure of the Anorexia Nervosa Inventory for Self-Rating in a population-based sample and derivation of a shortened form. Rathner G, Rainer B.	Unrelated
127.	Child Abuse Negl. 1999 Nov;23:1117-26. doi:10.1016/s0145-2134(99)00078-2. Prevalence and effects of child sexual abuse in a poor, rural community in El Salvador: a retrospective study of women after 12 years of civil war. Barthauer LM, Leventhal JM.	Unrelated
128.	Nihon Arukoru Yakubutsu Igakkai Zasshi. 2004 Dec;39:511-36. [Research on the basic attributes and psychosocial factors of alcoholics and their families in relation with prognosis after a year of treatment]. [Article in Japanese]. Nishikawa K.	Unrelated
129.	Psychiatr Pol. 1998 Mar-Apr;32:165-75. [Multifactorial aspects of eating disorders]. [Article in Polish] Banaś A, Januszkiewicz-Grabias A, Radziwiłłowicz P.	No OCD

130.	Wronski, M.-L.; Kuja-Halkola, R.; Hedlund, E.; Martini, M. I.; Lichtenstein, P.; Lundström, S.; Larsson, H.; Taylor, M. J.; Micali, N.; Bulik, C. M.; Dinkler, L. Mental and somatic conditions in children with the broad avoidant restrictive food intake disorder phenotype. JAMA Pediatrics, Vol 179(4), 2025, pp. 428–437. https://doi.org/10.1001/jamapediatrics.2024.6065	Included (duplicate)
131.	Tempia Valenta, S.; Beghelli, V.; Marcolini, F.; Rosinska, M.; De Ronchi, D.; Fernandez-Aranda, F.; Atti, A. R. Emotional dysregulation, obsessive compulsive traits, and eating disorders: Three constructs for one spectrum? Journal of Endocrinological Investigation, - 2025, [Online ahead of print]. https://doi.org/10.1007/s40618-025-02617-1	No ARFID
132.	Fodstad, J. C.; Russell, R.; Bullington, M.; Jones, L. B.; Iticovici, M.; Meudt, E. Treating obsessive compulsive disorder in adolescents and adults with Down syndrome: Results from a scoping rapid review. Journal of Autism and Developmental Disorders, Vol 55(5), 2025, pp. 1745–1753. https://doi.org/10.1007/s10803-024-06336-z	Unrelated
133.	Velimirović, M.; Robison, M.; Abber, S.; Duffy, A.; Rienecke, R. D.; Manwaring, J.; Blalock, D. V.; Riddle, M.; Mehler, P. S.; Joiner, T. E. Anxiety, obsessive-compulsive, and depressive symptom presentation and change throughout routine eating disorder treatment. European Eating Disorders Review, Vol 33(3), 2025, pp. 490–502. https://doi.org/10.1002/erv.3160	Included (duplicate)
134.	Tahir, M.; Zahid, A.; Afzal, S. Trapped by the arrows: Avoidant/restrictive food intake disorder and the illusion of control in type 1 diabetes mellitus. Cureus, Vol 17(6), 2025, e85539. https://doi.org/10.7759/cureus.85539	Unrelated
135.	Li, R.; Radhakrishnan, V. A case of avoidant/restrictive food intake disorder in an adult with schizophrenia and obsessive-compulsive disorder. Journal of the Academy of Consultation-Liaison Psychiatry, Vol 66(3), 2025, pp. 268–269. https://doi.org/10.1016/j.jaclp.2025.01.005	Case Report
136.	Wilson, D.; Krishnamorthy, G.; Mendes, R. A.; Withington, T.; Dalton, M.; Loxton, N. J. A comparison of psychiatric comorbid symptomology between adolescents with restrictive/avoidant food intake disorder, anorexia nervosa and atypical anorexia nervosa. European Eating Disorders Review, 2025, [Online ahead of print]. https://doi.org/10.1002/erv.70014	No OCD
137.	Viticchi, G.; Falsetti, L.; Di Felice, C.; De Vanna, G.; Salvemini, S.; Bartolini, M.; Moroncini, G.; Silvestrini, M. Variation in subtypes of obsessive-compulsive traits in migraine patients undergoing Onabotulinum Toxin A therapy. Toxins, Vol 17(4), 2025, art. 199. https://doi.org/10.3390/toxins17040199	Unrelated
138.	Clarke, A. T.; Fineberg, N. A.; Pellegrini, L.; Leuzzi, R.; Laws, K. R. From lockdown to liberation: How inflexible thinking, obsessive-compulsive and affective symptoms shape pandemic adjustment. Journal of Psychiatric Research, Vol 187, 2025, pp. 238–247. https://doi.org/10.1016/j.jpsychires.2025.05.003	Unrelated
139.	Retzler, C.; Retzler, J. Brief report: Local-global processing and co-occurrence of anxiety, autistic and obsessive-compulsive traits in a non-clinical sample. Journal of Autism and Developmental Disorders, Vol 55(2), 2025, pp. 764–771. https://doi.org/10.1007/s10803-022-05886-4	Unrelated
140.	Eddy, C. M. Self–other distinction and schizotypy: Affect sharing and alexithymia in the prediction of socially anxious and avoidant traits. Personality Disorders: Theory, Research, and Treatment, Vol 16(2), 2025, pp. 137–147. https://doi.org/10.1037/per0000669	Unrelated
141.	Marchetto, C.; Criscuolo, M.; Croci, I.; Bucci, M. E.; Caramadre, A.; Castiglioni, M. C.; Vicari, S.; Zanna, V. Family functioning and eating psychopathology in developmental restrictive eating disorders after Covid-19 lockdown. Eating and Weight Disorders, Vol 30(1), 2025, art. no. 36. https://doi.org/10.1007/s40519-025-01749-w	Unrelated

142.	Kapphahn, C.; Peet, B.; Gao, J.; Chan, A.; Farhadian, B.; Ma, M.; Silverman, M.; Tran, P.; Schlenk, N.; Thienemann, M.; Frankovich, J. Sudden onset disordered eating behaviors and appetite issues in a local clinical cohort of children with pediatric acute-onset neuropsychiatric syndrome (PANS). International Journal of Eating Disorders, Vol 58(7), 2025, pp. 1219–1232. https://doi.org/10.1002/eat.24388	Unrelated
143.	Mori, Y.; Suzuki, Y.; Miura, I. Long-acting injectable paliperidone palmitate for severe anorexia nervosa and comorbid autism spectrum disorder: A case report. Psychiatry and Clinical Neurosciences Reports, Vol 4(3), 2025, e70161. https://doi.org/10.1002/pcn5.70161	Unrelated
144.	Çınaroğlu M, Yılmazer E. Muscle Dysmorphia, Obsessive-Compulsive Traits, and Anabolic Steroid Use: A Systematic Review and Meta-Analysis. Behav Sci (Basel). 2025;15(9):1206. doi: 10.3390/bs15091206. PMID: 41009236; PMCID: PMC12466485.	Unrelated
145.	Kaçar AŞ, Balcı F. Perceptual decision making and metacognition in relation to obsessive-compulsive traits. Conscious Cogn. 2025 Nov;136:103944. doi: 10.1016/j.concog.2025.103944. Epub 2025 Oct 15. PMID: 41100960.	Unrelated
146.	Moccia L, Anesini MB, Callovini T, Janiri D, Policola C, Cintoni M, Focà F, Kotzalidis GD, Conti F, Bandettini Di Poggio A, Camardese G, Sani G. Understanding Orthorexia Nervosa: A Systematic Review of Meta-analytical Findings. Curr Nutr Rep. 2025 Dec 16;14(1):126. doi: 10.1007/s13668-025-00714-4. PMID: 41400796; PMCID: PMC12708686.	Unrelated
147.	Kucukterzi-Ali S, Ludlow AK, Gutierrez R, Fineberg NA, Gale TM. Pathological Eating Patterns in Adults Displaying Obsessive-Compulsive Symptoms: A Scoping Review. Eur Eat Disord Rev. 2025 Dec 23. doi: 10.1002/erv.70071. Epub ahead of print. PMID: 41437574.	No ARFID
148.	Ricci F, Valentini CP, Torales J, Caycho-Rodríguez T, Hualparuca-Olivera L, Castaldelli-Maia JM, Ventriglio A. When imagination turns into disorder: the case of maladaptive daydreaming. Int Rev Psychiatry. 2025 Sep–Nov;37(6–7):706–718. doi: 10.1080/09540261.2025.2562185. Epub 2025 Sep 18. PMID: 40965365.	Unrelated
149.	Halaç E, Ermis C, Gundogan N, Sut E, Turan S, Tunctürk M, Pekcanlar Akay A. Neurocognitive and behavioral characteristics of ADHD with cognitive disengagement syndrome and specific learning disorder. Appl Neuropsychol Child. 2025 Sep 24:1-10. doi: 10.1080/21622965.2025.2565430. Epub ahead of print. PMID: 40992776.	Unrelated
150.	Antonyan L, Shaheen SM, Burton CL, Baldwin G, Neill R, Easter P, MacMaster F, Hanna GL, Rosenberg D, Arnold PD. Association of Brain Structural Measurements and Polygenic Risk Scores with Obsessive-Compulsive Symptoms in Adolescents Diagnosed with Obsessive-Compulsive Disorder, Attention-Deficit/Hyperactivity Disorder, Anxiety, Depression, Autism and Tic Disorders. medRxiv [Preprint]. 2025 Dec 22:2025.12.17.25342484. doi: 10.64898/2025.12.17.25342484. PMID: 41480019; PMCID: PMC12755272.	Unrelated
	Search from PSYCINFO	
1.	A meta-analysis examining the relations among pathological gambling, obsessive-compulsive disorder, and obsessive-compulsive traits. Durdle,	No ARFID

	Heather; Gorey, Kevin M.; Stewart, Sherry H.;Psychological Reports, Vol 103(2), Oct, 2008 pp. 485-498.	
2.	A symptom network model of misophonia: From heightened sensory sensitivity to clinical comorbidity. Andermane, Nora;Bauer, Mathilde; Simner, Julia; Ward, Jamie; Journal of Clinical Psychology, Vol 79(10), Oct, 2023 pp. 2364-2387.	Unrelated
3.	Adult picky eaters with symptoms of avoidant/restrictive food intake disorder: Comparable distress and comorbidity but different eating behaviors compared to those with disordered eating symptoms. Zickgraf, Hana F.; Franklin, Martin E.; Rozin, Paul; Journal of Eating Disorders, Vol 4, Oct 29, 2016 ArtID: 26.	Included (duplicate)
4.	Adult picky eating. Phenomenology, taste sensitivity, and psychological correlates. Kauer, Jane; Pelchat, Marcia L.; Rozin, Paul; Zickgraf, Hana F.; Appetite, Vol 90, Jul 1, 2015 pp. 219-228.	No ARFID
5.	Age at first sexual activity: Clinical and cognitive associations. Harries, Michael D.; Paglia, Helen A.; Redden, Sarah A.; Grant, Jon E.; Annals of Clinical Psychiatry, Vol 30(2), May, 2018 pp. 102-112.	Unrelated
6.	Altered default mode network functional connectivity in individuals with co-occurrence of schizotypy and obsessive-compulsive traits. Wang, Yong-ming; Cai, Xin-lu; Zhou, Han-yu; Zhang, Rui-ting; Zhang, Yi-jing; Wang, Yan-yu; Cheung, Eric F. C.; Chan, Raymond C. K.; Psychiatry Research: Neuroimaging, Vol 305, Nov 30, 2020 ArtID: 111170.	Unrelated
7.	An unhealthy health behavior: Analysis of orthorexic tendencies among Hungarian gym attendees. Bóna, Enikő; Szél, Zsuzsanna; Kiss, Dániel; Gyarmathy, V. Anna; Eating and Weight Disorders, Vol 24(1), Feb 13, 2019 pp. 13-20.	Unrelated
8.	Anxiety, obsessive-compulsive, and depressive symptom presentation and change throughout routine eating disorder treatment. Velimirović, Mina; Robison, Morgan; Abber, Sophie; Duffy, Alan; Rienecke, Renee D.; Manwaring, Jamie; Blalock, Dan V.; Riddle, Megan; Mehler, Philip S.; Joiner, Thomas E.; European Eating Disorders Review Publisher	Lumping
9.	Anxious and obsessive-compulsive traits are independently associated with valuation of noninstrumental information. Bennett, Daniel; Sutcliffe, Kiran; Tan, Nicholas Poh-Jie; Smillie, Luke D.; Bode, Stefan; Journal of Experimental Psychology: General, Vol 150(4), Apr, 2021 pp. 739-755.	No ARFID
10.	Are the obsessive-compulsive traits a moderator for the relationship between autism and anorexia? A cross-sectional study among university students. Acikel, S. Burak; Cikili, Yahya; Journal of American College Health Publisher	Unrelated
11.	Brief report: Local–global processing and co-occurrence of anxiety, autistic and obsessive–compulsive traits in a non-clinical sample. Retzler, Chris; Retzler, Jenny; Journal of Autism and Developmental Disorders Publisher	Unrelated
12.	Characteristics of children with autism and unspecified intellectual developmental disorder (intellectual disability) presenting with severe self-	Unrelated

	injurious behaviours. Fong, Alison; Friedlander, Robin; Richardson, Anamaria; Allen, Katie; Zhang, Qian; International Journal of Developmental Disabilities, Vol 70(3), May, 2024 pp. 518-529.	
13.	Childhood obsessive–compulsive traits in anorexia nervosa patients, their unaffected sisters and healthy controls: A retrospective study. Degortes, Daniela; Zanetti, Tatiana; Tenconi, Elena; Santonastaso, Paolo; Favaro, Angela; European Eating Disorders Review, Vol 22(4), Jul, 2014 pp. 237-242.	Unrelated
14.	Communal nesting differentially attenuates the impact of pre-weaning social isolation on behavior in male and female rats during adolescence and adulthood. Bratzu, Jessica; Ciscato, Maria; Pisanu, Augusta; Talani, Giuseppe; Frau, Roberto; Porcu, Patrizia; Diana, Marco; Fumagalli, Fabio; Romualdi, Patrizia; Rullo, Laura; Trezza, Viviana; Ciccocioppo, Roberto; Sanna, Fabrizio; Fattore, Liana; Frontiers in Behavioral Neuroscience, Vol 17, Oct 17, 2023	Unrelated
15.	Corticostriatal-limbic correlates of sub-clinical obsessive-compulsive traits. Kubota, Yasutaka; Sato, Wataru; Kochiyama, Takanori; Uono, Shota; Yoshimura, Sayaka; Sawada, Reiko; Toichi, Motomi; Psychiatry Research: Neuroimaging, Vol 285, Mar 30, 2019 pp. 40-46.	Unrelated
16.	COVID-19 pandemic and mental health in Lebanon: A cross-sectional study. El Othman, Radwan; Touma, Elsie; El Othman, Rola; Haddad, Chadia; Hallit, Rabih; Obeid, Sahar; Salameh, Pascale; Hallit, Souheil; International Journal of Psychiatry in Clinical Practice, Vol 25(2), Jun, 2021 pp. 152-163.	Unrelated
17.	Differential comorbidity profiles in avoidant/restrictive food intake disorder and anorexia nervosa: Does age play a role? Kambanis, P. Evelyna; Harshman, Stephanie G.; Kuhnle, Megan C.; Kahn, Danielle L.; Dreier, Melissa J.; Hauser, Kristine; Slattery, Meghan; Becker, Kendra R.; Breithaupt, Lauren; Misra, Madhusmita; Micali, Nadia; Lawson, Elizabeth A.; Eddy, Kamryn T.; Thomas, Jennifer J.; International Journal of Eating Disorders, Vol 55(10), Oct, 2022 pp. 1397-1403.	Lumping
18.	Disentangling orthorexia nervosa from healthy eating and other eating disorder symptoms: Relationships with clinical impairment, comorbidity, and self-reported food choices. Zickgraf, Hana F.; Ellis, Jordan M.; Essayli, Jamal H.; Appetite, Vol 134, Mar 1, 2019 pp. 40-49.	Unrelated
19.	Disordered eating and food restrictions in children with PANDAS/PANS. Toufexis, Megan D.; Hommer, Rebecca; Gerardi, Diana M.; Grant, Paul; Rothschild, Leah; D'Souza, Precilla; Williams, Kyle; Leckman, James; Swedo, Susan E.; Murphy, Tanya K.; Journal of Child and Adolescent Psychopharmacology, Vol 25(1), Feb, 2015 Special Issue: Pediatric Acute-Onset Neuropsychiatric Syndrome. pp. 48-56. Publisher: Mary Ann Liebert, Inc.; [Journal Article];	Unrelated
20.	Dysfunctional personality beliefs and executive performance in patients with juvenile myoclonic epilepsy. Taura, Mariângela; Gama, André P.; Sousa, Artur V. M.; Noffs, Maria Helena S.; Alonso, Neide B.; Yacubian, Elza M.; Guilhoto, Laura M.; Epilepsy & Behavior, Vol 105, Apr, 2020 ArtID: 106958.	Unrelated

21.	Eating behaviours and personality characteristics of clinicians and researchers working in eating disorders. Poiani-Cordella, Catiray; Toh, Wei Lin; Phillipou, Andrea; Eating Disorders: The Journal of Treatment & Prevention, Vol 32(2), Mar-Apr, 2024 pp. 195-211.	Unrelated
22.	Eating disorders in the postpartum period. Sutandar, Kalam; *In:* Postpartum mental health disorders: A casebook. Robinson, Gail Erlick (Ed); Nadelson, Carol C. (Ed); Gisele, Apter (Ed); Publisher: Oxford University Press; 2020, pp. 89-97.	Unrelated
23.	ERN as a transdiagnostic marker of the internalizing-externalizing spectrum: A dissociable meta-analytic effect. Pasion, Rita; Barbosa, Fernando; Neuroscience and Biobehavioral Reviews, Vol 103, Aug, 2019 pp. 133-149.	Unrelated
24.	Fear of vomiting and low body weight in two pediatric patients: Diagnostic challenges. Maertens, Charlotte; Couturier, Jennifer; Grant, Christina; Johnson, Natasha; Journal of the Canadian Academy of Child and Adolescent Psychiatry / Journal de l'Académie canadienne de psychiatrie de l'enfant et de l'adolescent, Vol 26(1), Win 2017 pp. 59-61.	Unrelated
25.	Heterozygosity for neuronal ceroid lipofuscinosis predisposes to bipolar disorder. Privitera, Flavia; Trusso, Maria A.; Valentino, Floriana; Doddato, Gabriella; Fallerini, Chiara; Brunelli, Giulia; D'Aurizio, Romina; Furini, Simone; Goracci, Arianna; Fagiolini, Andrea; Mari, Francesca; Renieri, Alessandra; Ariani, Francesca; Brazilian Journal of Psychiatry, Vol 45(1), Jan-Feb, 2023 pp. 11-19.	Unrelated
26.	How untidiness moves the motor system. Fiori, Francesca; Ciricugno, Andrea; Rusconi, Maria Luisa; Slaby, Ryan J.; Cattaneo, Zaira; Perceptual and Motor Skills, Vol 129(3), Jun, 2022 pp. 399-414.	Unrelated
27.	Individual obsessive-compulsive traits are associated with poorer adjustment to the easing of COVID-19 restrictions. Fineberg, Naomi A.; Pellegrini, Luca; Burkauskas, Julius; Clarke, Aaron; Laws, Keith R.; Journal of Psychiatric Research, Vol 148, Apr, 2022 pp. 21-26.	Unrelated
28.	Metacognitive interpersonal therapy in groups for over-regulated personality disorders: A single case study. Popolo, Raffaele; MacBeth, Angus; Canfora, Flaviano; Rebecchi, Daniela; Toselli, Cecilia; Salvatore, Giampaolo; Dimaggio, Giancarlo; Journal of Contemporary Psychotherapy: On the Cutting Edge of Modern Developments in Psychotherapy, Vol 49(1), Mar 15, 2019 pp. 49-59.	Unrelated
29.	Mice lacking Ptprd exhibit deficits in goaldirected behavior and female-specific impairments in sensorimotor gating. Ho, Emily V.; Welch, Amanda; Thompson, Summer L.; Knowles, James A.; Dulawa, Stephanie C.; PLoS ONE, Vol 18(5), May 19, 2023 ArtID: e0277446.	Unrelated
30.	Network analysis of schizotypal personality traits and their association with other subclinical psychiatric features. Zhang, Rui-ting; Zhou, Han-yu; Wang, Yong-ming; Yang, Zhuo-ya; Wang, Yi; So, Suzanne H.; Chiu, Chui-De; Leung, Patrick W. L.; Cheung, Eric F. C.; Chan, Raymond C. K.; Asian Journal of Psychiatry, Vol 44, Aug, 2019 pp. 209-216.	Unrelated

31.	Network structure of anticipatory pleasure and risk features: Evidence from a large college sample. Zhang, Rui-ting; Wang, Yi; Yang, Zhuo-ya; Li, Ying; Wang, Yong-ming; Cheung, Eric F. C.; Shum, David H. K.; Yang, Tian-Xiao; Barkus, Emma J.; Chan, Raymond C. K.; PsyCh Journal, Vol 9(2), Apr, 2020 pp. 223-233.	Unrelated
32.	Obsessive–compulsive traits in children and adolescents with Asperger syndrome. Ruta, Liliana; Mugno, Diego; D'Arrigo, Valentina Genitori; Vitiello, Benedetto; Mazzone, Luigi; European Child & Adolescent Psychiatry, Vol 19(1), Jan, 2010 pp. 17-24.	No ARFID
33.	Orthorexia nervosa: A review of psychosocial risk factors. McComb, Sarah E.; Mills, Jennifer S.; Appetite, Vol 140, Sep 1, 2019 pp. 50-75.	Review
34.	Overlap of obsessive-compulsive traits and autistic traits in college students. Tang, Su-qin; Wang, Jian-ping; Liu, Jun; Sun, Hong-wei; Tang, Tan; Chinese Journal of Clinical Psychology, Vol 20(3), Jun, 2012 pp. 353-355.	No ARFID
35.	Oversensitivity and overgeneralization of the error withdrawal response in different obsessive-compulsive traits. Hochman, Eldad Yitzhak; Tal, Liron; Current Psychology: A Journal for Diverse Perspectives on Diverse Psychological Issues, Vol 39(4), Aug, 2020 pp. 1314-1321.	Unrelated
36.	Personality differences between diocesan and religious order catholic priests with alcohol use disorder. Richardson, Emily Marie; Dissertation Abstracts International: Section B: The Sciences and Engineering, Vol 84(12-B).	Unrelated
37.	Quality of life and infertility stress in homologous and heterologous medically assisted reproduction: The role of common and specific psychopathological traits. Pozza, Andrea; Dèttore, Davide; Coccia, Maria E.; Perspectives in Psychiatric Care, Vol 57(2), Apr, 2021 pp. 717-725.	Unrelated
38.	Relationships between compulsive exercise, quality of life, psychological distress and motivation to change in adults with anorexia nervosa. Young, Sarah; Touyz, Stephen; Meyer, Caroline; Arcelus, Jon; Rhodes, Paul; Madden, Sloane; Pike, Kathleen; Attia, Evelyn; Crosby, Ross D.; Hay, Phillipa; Journal of Eating Disorders, Vol 6, Feb 5, 2018 ArtID: 2.	Unrelated
39.	Rigidity and sensory sensitivity: Independent contributions to selective eating in children, adolescents, and young adults. Zickgraf, Hana F.; Richard, Emily; Zucker, Nancy L.; Wallace, Gregory L.; Journal of Clinical Child and Adolescent Psychology, Vol 51(5), Sep-Oct, 2022 pp. 675-687.	No ARFID
40.	Schizotypal and obsessive–compulsive traits: Co-occurrence rate and relationship with executive function, emotion experience, and emotion expressivity in college students. Shan, Hai-di; Zhang, Rui-ting; Jiang, Shu-yao; Wang, Yong-ming; Liu, Ya-fei; Cheung, Eric F. C.; Chan, Raymond C. K.; PsyCh Journal, Vol 9(5), Oct, 2020 pp. 749-759.	Unrelated
41.	Serotonin and love: Supporting evidence from a patient suffering from obsessive-compulsive disorder. Marazziti, Donatella; Stahl, Stephen M.; Journal of Clinical Psychopharmacology, Vol 38(1), Feb, 2018 pp. 99-101.	Unrelated

42.	Serotonin system genes and hoarding with and without other obsessive–compulsive traits in a population-based, pediatric sample: A genetic association study. Sinopoli, Vanessa M.; Erdman, Lauren; Burton, Christie L.; Park, Laura S.; Dupuis, Annie; Shan, Janet; Goodale, Tara; Shaheen, S-M.; Crosbie, Jennifer; Schachar, Russell J.; Arnold, Paul D.; Depression and Anxiety, Vol 37(8), Aug, 2020 pp. 760-770.	Unrelated
43.	Suppress or accept? A pilot study to evaluate the effect of coping strategies on ERN amplitude among individuals with obsessive-compulsive traits. Zambrano-Vazquez, Laura; Szabo, Yvette Z.; Santerre, Craig Lee; Allen, John J. B.; Acta Neuropsychologica, Vol 17(3), 2019 pp. 283-301.	Unrelated
44.	The 'Resilient Brain': Challenging key characteristics associated with the concept of resilience. Savulich, George; Ferry-Bolder, Eve; Lim, Tsen Vei; Mak, Elijah; Ersche, Karen D.; Psychological Medicine, Vol 53(14), Oct, 2023 pp. 6933-6936.	Unrelated
45.	The correlation between peripheral blood micro-ribonucleic acid expression level and personality disorder in patients with schizophrenia. Wei, Honghui; Kong, Lingming; Zhu, Xiaoli; Chen, Shengdong; Zhang, Liyi; Niu, Wei; Alpha Psychiatry, Vol 25(1), Feb, 2024 pp. 23-29.	Unrelated
46.	The moderating role of race/ethnicity and cultural factors on the relationship between internalized stigma, discrimination and help-seeking attitudes. Martinez de Andino, Ana; Dissertation Abstracts International Section A: Humanities and Social Sciences, Vol 81(5-A).	Unrelated
47.	The Toronto Obsessive-Compulsive Scale: Psychometrics of a dimensional measure of obsessive-compulsive traits. Park, Laura S.; Burton, Christie L.; Dupuis, Annie; Shan, Janet; Storch, Eric A.; Crosbie, Jennifer; Schachar, Russell J.; Arnold, Paul D.; Journal of the American Academy of Child & Adolescent Psychiatry, Vol 55(4), Apr, 2016 pp. 310-318.	Unrelated
48.	Treating obsessive compulsive disorder in adolescents and adults with down syndrome: Results from a scoping rapid review. Fodstad, Jill C.; Russell, Rachel; Bullington, Molly; Jones, Lauren B.; Iticovici, Micah; Meudt, Emily; Journal of Autism and Developmental Disorders	Review
49.	Validation of the Chinese Involuntary Musical Imagery Scale and its application in mainland China. Jue, Deng; Jianping, Ma; Yiduo, Ye; Musicae Scientiae, Vol 26(2), Jun, 2022 pp. 326-338.	Unrelated
50.	What process works for whom: Individual differences and the impact of therapy techniques and treatment mechanisms. Keefe, John Raymond; Dissertation Abstracts International Section A: Humanities and Social Sciences, Vol 81(6-A).	Unrelated
51.	A behavioral comparison of male and female adults with high functioning autism spectrum conditions. Lai, Meng-Chuan; Lombardo, Michael V.; Pasco, Greg; Ruigrok, Amber N.V.; Wheelwright, Sally J.; Sadek, Susan A.; Chakrabarti, Bhismadev; Baron-Cohen, Simon; MRC AIMS Consortium; PLoS ONE, Vol 6(6), Jun 13, 2011 ArtID: e20835.	Unrelated

52.	ADHD symptoms and insistence on sameness in Prader-Willi syndrome. Wigren, Margareta; Hansen, S.; Journal of Intellectual Disability Research, Vol 49(6), Jun, 2005 pp. 449-456.	Unrelated
53.	An autistic dimension: A proposed subtype of obsessive-compulsive disorder. Bejerot, Susanne; Autism, Vol 11(2), Mar, 2007 pp. 101-110.	Unrelated
54.	An examination of decision making in bulimia nervosa.Liao, Pei-Chi;Uher, Rudolf;Lawrence, Natalia;Treasure,Janet; Schmidt, Ulrike; Campbell, Iain C.; Collier, David A.;Tchanturia,Kate; Journal of Clinical and Experimental Neuropsychology, Vol 31(4), May, 2009 pp. 455-461.	Unrelated
55.	Association of Neuregulin 1 rs3924999 genotype with antisaccades and smooth pursuit eye movements. Schmechtig, A.; Vassos, E.; Kumari, V.; Hutton, S. B.; Collier, D. A.; Morris, R. G.; Williams, S. C. R.; Ettinger, U.; Genes, Brain & Behavior, Vol 9(6), Aug, 2010 pp. 621-627.	Unrelated
56.	Attentional process of emotional information: Comparison between clinical and nonclinical obsessive–compulsive disorder. Unoki, Keiko; Kasuga, Takashi; Matsushima, Eisuke; Ohta, Katsuya; Doi, Nagafumi; Seishin Igaku, Vol 42(3), Mar, 2000 pp. 273-280.	Unrelated
57.	Atypical anorexia nervosa is not related to brain structural changes in newly diagnosed adolescent patients. Olivo, Gaia; Dahlberg, Linda Solstrand; Wiemerslage, Lyle; Swenne, Ingemar; Zhukovsky, Christina; Salonen-Ros, Helena; Larsson, Elna-Marie; Gaudio, Santino; Brooks, Samantha J.; Schiöth, Helgi B.; International Journal of Eating Disorders, Vol 51(1), Jan, 2018 pp. 39-45.	Unrelated
58.	Avoidance and behavioural flexibility in obsessive compulsive disorder. Hassoulas, Athanasios; McHugh, Louise; Reed, Phil; Journal of Anxiety Disorders, Vol 28(2), Mar, 2014 pp. 148-153.	No ARFID
59.	Behavioural and trait changes in Parkinsonian patients with impulse control disorder after switching from dopamine agonist to levodopa therapy: results of REIN-PD trial. Lee, JeeYoung; Jeon, Beomseok; Koh, Seong-Beom; Yoon, Won Tae; Lee, Ho-Won; Kwon, Oh Dae; Kim, Jae Woo; Kim, Jong-Min; Ma, Hyeo-Il; Kim, Hee-Tae; Baik, Jong Sam; Cho, Jinwhan; REIN-PD Investigators; Journal of Neurology, Neurosurgery & Psychiatry, Vol 90(1), Jan, 2019 pp. 30-37.	Unrelated
60.	Body dysmorphic traits and personality disorder patterns in rhinoplasty seekers. Barahmand, Usha; Mozdsetan, Nasrin; Narimani, Mohammad; Asian Journal of Psychiatry, Vol 3(4), Dec, 2010 pp. 194-199.	Unrelated
61.	Clinical Reasoning: A 16-year-old girl with subacute weakness and sensory loss. Quintanilla-Bordás, Carlos; Nourbakhsh, Bardia; Strober, Jonathan; Raffel, Corey; Waubant, Emmanuelle; Neurology, Vol 88(23), Jun 6, 2017 pp. e225-e229.	Unrelated
62.	Cluster C personality accentuation in patients with takotsubo cardiomyopathy: Personality traits mark vulnerability. Müller, Helge H.; Sperling, Wolfgang; Kornhuber, Johannes; Psychiatria Danubina, Vol 29(1), 2017 pp. 93.	Unrelated

63.	Comparing two short forms of the Hewitt–Flett Multidimensional Perfectionism Scale. Stoeber, Joachim; Assessment, Vol 25(5), Jul, 2018 pp. 578-588.	Unrelated
64.	Compulsive exercise: Links, risks and challenges faced.Lichtenstein, Mia Beck; Hinze, Cecilie Juul; Emborg, Bolette; Thomsen, Freja; Hemmingsen, Simone Daugaard; Psychology Research and Behavior Management, Vol 10, Mar 30, 2017 ArtID: 85-95.	Unrelated
65.	Co-occurrence of obsessive-compulsive personality traits in young and middle-aged Japanese alcohol-dependent men. Suzuki, Kenji; Muramatsu, Taro; Takeda, Aya; Shirakura, Katsuyuki; Alcoholism: Clinical and Experimental Research, Vol 26(8), Aug, 2002 pp. 1223-1227.	Unrelated
66.	Correlation between eating disorder and obsessive-compulsive disorder in adolescents. Chappa, Herbert J.; Revista Argentina de Clínica Psicológica, Vol 21(2), Aug, 2012 Special Issue: Up-date in eating disorders. pp. 113-119.	Lumping
67.	Do the traits of autism-spectrum overlap with those of schizophrenia or obsessive-compulsive disorder in the general population? Wakabayashi, Akio; Baron-Cohen, Simon; Ashwin, Chris; Research in Autism Spectrum Disorders, Vol 6(2), Apr-Jun, 2012 pp. 717-725.	Unrelated
68.	Eating disorders. Cook-Cottone, Catherine; Smith, Amanda; *In:* The neuropsychology of psychopathology. Noggle, Chad A. (Ed); Dean, Raymond S. (Ed); Publisher: Springer Publishing Company; 2013, pp. 441-456.	No OCD
69.	Epilepsy and obsessive-compulsive disorder. Nishida, Takuji; Sugiyama, Osamu; Inoue, Yushi; Japanese Journal of Child and Adolescent Psychiatry, Vol 47(2), 2006 pp. 154-158.	Unrelated
70.	Evaluation, treatment, and monitoring of disruptive physician behavior. Harmon, Larry;Pomm, Raymond M.; Psychiatric Annals, Vol 34(10), Oct, 2004 pp. 770-774.	Unrelated
71.	Evidence for a genetic overlap between body dysmorphic concerns and obsessive–compulsive symptoms in an adult female community twin sample. Monzani, Benedetta; Rijsdijk, Fruhling; Iervolino, Alessandra C.; Anson, Martin; Cherkas, Lynn; Mataix-Cols, David; American Journal of Medical Genetics Part B: Neuropsychiatric Genetics, Vol 159B(4), Jun, 2012 pp. 376-382.	Unrelated
72.	Hoarding disorder: A study of a general Italian population based sample. Bottesi, Gioia; Novara, Caterina; Psicoterapia Cognitiva e Comportamentale, Vol 18(2), 2012 pp. 141-156.	Unrelated
73.	Impulsive and compulsive traits in eating disordered patients compared with controls. Claes, Laurence; Vandereycken, Walter; Vertommen, Hans; Personality and Individual Differences, Vol 32(4), Mar, 2002 pp. 707-714.	Lumping
74.	Increased prevalence of nocturnal smoking in restless legs syndrome (RLS). Provini, F.; Antelmi, E.; Vignatelli, L.; Zaniboni, A.; Naldi, G.; Calandra-Buonaura, G.; Vetrugno, R.; Plazzi, G.; Pizza, F.; Montagna, P.; Sleep Medicine, Vol 11(2), Feb, 2010 pp. 218-220.	Unrelated

75.	Influence of religious aspects and personal beliefs on psychological behavior: Focus on anxiety disorders. Agorastos, Agorastos; Demiralay, Cüneyt; Huber, Christian G.; Psychology Research and Behavior Management, Vol 7, Mar 10, 2014 ArtID: 93-101.	Unrelated
76.	Initial treatment success followed by failure: How do we deal with toxic core beliefs in the context of comorbid major depression with psychotic features, social phobia, and axis II pathology? Prisciandaro, James J.; Roberts, John E.; Clinical Case Studies, Vol 8(3), Jun, 2009 pp. 193-209.	Unrelated
77.	Job involvement, obsessive-compulsive personality traits, and workaholic behavioral tendencies. Mudrack, Peter E.; Journal of Organizational Change Management, Vol 17(5), 2004 pp. 490-508.	Unrelated
78.	Lifetime course of eating disorders: Design and validity testing of a new strategy to define the eating disorders phenotype. Anderluh, M.; Tchanturia, K.; Rabe-Hesketh, S.; Collier, D.; Treasure, J.; Psychological Medicine, Vol 39(1), Jan, 2009 pp. 105-114.	No OCD
79.	Maladaptive personality traits increase subjectively during the course of schizophrenia spectrum disorders. Schroeder, Katrin; Naber, Dieter; Huber, Christian G.; Journal of Nervous and Mental Disease, Vol 202(4), Apr, 2014 pp. 319-323.	Unrelated
80.	Medical and psychiatric morbidity in obese women with and without binge eating. Bulik, Cynthia M.; Sullivan, Patrick F.; Kendler, Kenneth S.; International Journal of Eating Disorders, Vol 32(1), Jul, 2002 pp. 72-78.	Unrelated
81.	Migraine headache in patients with Tourette syndrome. Kwak, Carolyn; Vuong, Kevin Dat; Jankovic, Joseph; Archives of Neurology, Vol 60(11), Nov, 2003 Special Issue: Pain 2003. pp. 1595-1598.	Unrelated
82.	Mood-congruent and mood-incongruent psychotic symptoms in major depression: The role of severity and personality. Tonna, Matteo; De Panfilis, Chiara; Marchesi, Carlo; Journal of Affective Disorders, Vol 141(2-3), Dec 10, 2012 pp. 464-468.	Unrelated
83.	Nitrite inhalant abuse in antisocial youth: Prevalence, patterns, and predictors. Hall, Martin T.; Howard, Matthew O.; Journal of Psychoactive Drugs, Vol 41(2), Jun, 2009 pp. 135-143.	Unrelated
84.	Obsessive and compulsive traits in pervasive developmental disorder. Toichi, Motomi; Japanese Journal of Child and Adolescent Psychiatry, Vol 47(2), 2006 pp. 127-134.	No ARFID
85.	Obsessive compulsive disorder: Psychoanalytic and neuropsychological interface. Mukhopadhyay, Pritha; Tarafder, Sreemoyee; *In:* Obsessive compulsive disorder: A neuropsychological approach. Mukhopadhyay, Pritha (Ed); Tarafder, Sreemoyee (Ed).	No ARFID
86.	Obsessive compulsive traits and symptoms in family members of autistic individuals. Harden, Susan Patricia; Dissertation Abstracts International: Section B: The Sciences and Engineering, Vol 63(2-B) pp. 1027.	Unrelated

87.	Obsessive-compulsive behaviors in parents of multiplex autism families. Hollander, Eric; King, Audrey; Delaney, Katherine; Smith, Christopher J.; Silverman, Jeremy M.; Psychiatry Research, Vol 117(1), Jan, 2003 pp. 11-16.	Unrelated
88.	Obsessive–compulsive personality traits in youth with obsessive–compulsive disorder. Park, Jennifer M.; Storch, Eric A.; Pinto, Anthony; Lewin, Adam B.; Child Psychiatry and Human Development, Vol 47(2), Apr, 2016 pp. 281-290.	Unrelated
89.	Obsessive-compulsive symptoms in Parkinson's disease. Alegret, M.; Junqué, C.; Valldeoriola, F.; Vendrell, P.; Martí, M. J.; Tolosa, E.; Journal of Neurology, Neurosurgery & Psychiatry, Vol 70(3), Mar, 2001 pp. 394-396.	Unrelated
90.	Personality development characteristics of women with anorexia nervosa, their healthy siblings and healthy controls: What prevents and what relates to psychopathology? Amianto, Federico; Abbate-Daga, Giovanni; Morando, Sara; Sobrero, Cinzia; Fassino, Secondo; Psychiatry Research, Vol 187(3), May 30, 2011 pp. 401-408.	Unrelated
91.	Personality dimensions and cognitive functioning of relatives of persons diagnosed with schizophrenia and Bipolar I disorder: A comparative and predictive study. Paavola, Julie; Dissertation Abstracts International: Section B: The Sciences and Engineering, Vol 75(7-B)(E).	Unrelated
92.	Psychological functioning measures in patients with primary insomnia and sleep state misperception. Dittoni, S.; Mazza, M.; Losurdo, A.; Testani, E.; Giacopo, R.; Marano, G.; Di Nicola, M.; Farina, B.; Mariotti, P.; Mazza, S.; Della Marca, G.; Acta Neurologica Scandinavica, Vol 128(1), Jul, 2013 pp. 54-60.	Unrelated
93.	Sense and sensibility: Relating behavior control issues with self-regulation of sensory input. Newby, Robert F.; Dunn, Winnie; *In:* Pediatric neuropsychology case studies: From the exceptional to the commonplace. Apps, Jennifer Niskala (Ed); Newby, Robert F. (Ed); Roberts, Laura Weiss (Ed).	Unrelated
94.	Set shifting in anorexia nervosa: An examination before and after weight gain, in full recovery and relationship to childhood and adult OCPD traits. Tchanturia, K.; Morris, R. G.; Anderluh, M. Brecelj; Collier, D. A.; Nikolaou, V.; Treasure, J.; Journal of Psychiatric Research, Vol 38(5), Sep-Oct, 2004 pp. 545-552.	Unrelated
95.	Single-victim and serial sexual homicide offenders: Differences in crime, paraphilias and personality traits. Chan, Heng Choon (Oliver); Beauregard, Eric; Myers, Wade C.; Criminal Behaviour and Mental Health, Vol 25(1), Feb, 2015 pp. 66-78.	Unrelated
96.	The case of Mrs. I. Huppertz, Bernd; *In:* Psychotherapy in the wake of war: Discovering multiple psychoanalytic traditions. Huppertz, Bernd (Ed); Publisher: Jason Aronson; 2013, pp. 41-46. [Chapter]	Unrelated
97.	The contribution of fear conditioning to pathological anxiety: An investigation of conditioned fear generalization in ocd traits and ptsd. Kaczkurkin, Antonia; Dissertation Abstracts International: Section B: The Sciences and Engineering, Vol 76(3-B)(E).	Unrelated

98.	The development of a psychological theory of needle fixation. Pates, R. M.; Gray, N.; Journal of Substance Use, Vol 14(5), Oct, 2009 pp. 312-324.	Unrelated
99.	The phenomenology of attention-deficit/hyperactivity disorder in Tourette syndrome. Rothenberger, Aribert; Roessner, Veit; *In:* Tourette syndrome. Martino, Davide (Ed); Leckman, James F. (Ed); Publisher: Oxford University Press; 2013, pp. 26-49.	Unrelated
100.	Incompleteness as a link between obsessive-compulsive personality traits and specific symptom dimensions of obsessive-compulsive disorder. Ecker, Willi; Gönner, Sascha; PTT: Persönlichkeitsstörungen Theorie und Therapie, Vol 11(2), Jun, 2007 pp. 111-122.	Unrelated
101.	A family study of personality manifestations in two highly genetic subtypes of mood disorder. Vuchetich, John Patrick; Dissertation Abstracts International: Section B: The Sciences and Engineering, Vol 59(4-B) pp. 1871.	Unrelated
102.	A psychiatric study of patients with regional enteritis. Ford, Charles V.; Glober, Gary A.; Castelnuovo-Tedesco, Pietro; JAMA: Journal of the American Medical Association, 208(2), 1969 pp. 311-315.	Unrelated
103.	A short clinical diagnostic self-rating scale for psychoneurotic patients: The Middlesex Hospital Questionnaire (M.H.Q.). Crown, Sidney; Crisp, A. H.; The British Journal of Psychiatry, 112(490), 1966 pp. 917-923.	Unrelated
104.	Anorexia nervosa: Demographic and clinical features in 94 cases. Halmi, Katherine A.; Psychosomatic Medicine, Vol. 36(1), Jan, 1974 pp. 18-26.	Unrelated
105.	Authors' reply. Alegret, M.; Junqué, C.; Vendrell, P.; Valldeoriola, F.; Marti, M. J.; Tolosa, E.; Journal of Neurology, Neurosurgery & Psychiatry, Vol 72(3), Mar, 2002 pp. 420-421.	Unrelated
106.	Cognitive-behavioral treatment of body dysmorphic disorder: A case report. Schmidt, Norman B.; Harrington, Patrick; Journal of Behavior Therapy and Experimental Psychiatry, Vol 26(2), Jun, 1995 pp. 161-167.	Case Report
107.	Comorbidity of obsessive-compulsive and anorexic behaviors in undergraduate females. Mulfinger, Amanda Margaret Marie; Dissertation Abstracts International: Section B: The Sciences and Engineering, Vol 68(7-B) pp. 4838.	No ARFID
108.	Comparison of demographic and clinical features in patient groups with different ages and weights at onset of anorexia nervosa. Halmi, Katherine A.; Journal of Nervous and Mental Disease, Vol. 158(3), Mar, 1974 pp. 222-225.	Unrelated
109.	Complaints of constipation in obsessive-compulsive disorder. North, Carol S.; Napier, Mark; Alpers, David H.; Spitznagel, Edward L.; Annals of Clinical Psychiatry, Vol 7(2), Jun, 1995 pp. 65-70.	Unrelated
110.	Defense mechanisms and personality disorders: An empirical test of Millon's theory. Berman, Stacey M. Whyne; McCann, Joseph T.; Journal of Personality Assessment, Vol 64(1), Feb, 1995 pp. 132-144.	Unrelated

111.	Do some cases of anorexia nervosa reflect underlying autistic-like conditions? Gillberg, C.; Råstam, M.; Behavioural Neurology, Vol 5(1), Mar, 1992 pp. 27-32.	Unrelated
112.	Dopamine agonist withdrawal syndrome in a patient with restless legs syndrome. Dorfman, Benjamin J.; Nirenberg, Melissa J.; Parkinsonism & Related Disorders, Vol 19(2), Feb, 2013 pp. 269-270.	Unrelated
113.	Dysfunctional thinking in anorexia and bulimia nervosa. Clark, David A.; Feldman, Joan; Channon, Shelley; Cognitive Therapy and Research, Vol 13(4), Aug, 1989 pp. 377-387.	Unrelated
114.	Eating attitudes and personality variables in a nonclinical sample. Pumariega, Andres J.; LaBarbera, Joseph D.; International Journal of Eating Disorders, Vol 5(2), Feb, 1986 pp. 285-294.	Lumping
115.	Eating disorder and obsessive-compulsive disorder: Neurochemical and phenomenological commonalities. Jarry, Josée L.; Vaccarino, Franco J.; Journal of Psychiatry & Neuroscience, Vol 21(1), Jan, 1996 pp. 36-48.	No ARFID
116.	Emotional inhibition in personality disorders. Popolo, Raffaele; Lysaker, Paul H.; Salvatore, Giampalo; Montano, Antonella; Buonocore, Luisa; Sirri, Laura; Imbimbo, Antonella; Dimaggio, Giancarlo; Psychotherapy and Psychosomatics, Vol 83(6), Nov, 2014 pp. 377-378.	Unrelated
117.	Exacerbation of Gilles de la Tourette's syndrome associated with thermal stress:A family study.Lombroso, Paul J.;Mack, G.;Scahill, L.;King, Robert A.;Leckman, J.F.;Neurology, Vol 41(12),Dec,1991 pp.1984-1987.	Unrelated
118.	Familial eating concerns and psychopathological traits: Causal implications of transgenerational effects.Steiger,Howard;Stotland,Stephen;Trottier, Julie; Ghadirian,A.M.; International Journal of Eating Disorders,Vol 19(2), Mar,1996 pp.147-157.	Unrelated
119.	Fluvoxamine in selective mutism. Lafferty, Julie E.; Constantino, John N.; Journal of the American Academy of Child & Adolescent Psychiatry, Vol 37(1), Jan, 1998 pp. 12-13.	Unrelated
120.	How I practice: Treating women golfers with eating disorders: Unique issues and concerns. Moore, Mary Ann; Eating Disorders: The Journal of Treatment & Prevention, Vol 7(3), Fal 1999 Special Issue: Athletes and eating disorders. pp. 245-248.	Unrelated
121.	MDA-assisted psychotherapy with neurotic outpatients: A pilot study. Yensen, Richard; Di Leo, Francesco B.; Rhead, John C.; Richards, William A.; Soskin, Robert A.; Turek, Brahim; Kurland, Albert A.; Journal of Nervous and Mental Disease, Vol 163(4), Oct, 1976 pp. 233-245.	Unrelated
122.	Neuropsychiatric and biological characteristics of X-linked MAO-A deficiency syndrome: A single case intervention study. Tuinier, S.; Verhoeven, W.M.A.; Scherders, M.J.W.T.; Fekkes, D.; et al; New Trends in Experimental & Clinical Psychiatry, Vol 11(4), Oct-Dec, 1995 pp. 99-107.	Unrelated
123.	Norms for the anorexia nervosa inventory for self-assessment in female adolescents in the risk group for eating disorders.Rathner, Günther; Rainer,	Unrelated

	Barbara; Zeitschrift für Klinische Psychologie, Psychiatrie und Psychotherapie, Vol 45(3), 1997 pp. 302-318.	
124.	Obsessive difficult temperament. Carey, William B.; Journal of the American Academy of Child & Adolescent Psychiatry, Vol 36(6), Jun, 1997 pp. 722.	No ARFID
125.	Obsessive-compulsive phenomenon and Parkinson's disease. Sharma, P.; Gupta, N.; Journal of Neurology, Neurosurgery & Psychiatry, Vol 72(3), Mar, 2002 pp. 420.	Unrelated
126.	Patterns of attachment and psychopathology in a sample of outpatient women. Peterson, Betty Taylor; Dissertation Abstracts International: Section B: The Sciences and Engineering, Vol 55(7-B) pp. 3037.	Unrelated
127.	Perceptual and cognitive factors in obsessive-compulsive behavior and the development of a computer-mounted personality instrument. Lambirth, Thomas T.; Dissertation Abstracts International, Vol 49(12-B, Pt 1) pp. 5523.	Unrelated
128.	Preadolescence and early adolescence: A reconstruction. Brockman, David Dean; *In:* Childhood bereavement and its aftermath. Altschul, Sol (Ed); Publisher: International Universities Press, Inc; 1988, pp. 351-376. [Chapter]	Unrelated
129.	Psychoanalytic profile of a creative mind: Eros and Thanatos in the life of Heinrich Schliemann. Niederland, W. G.; Psychotherapy and Psychosomatics, 15(2-4), 1967 pp. 200-219.	Unrelated
130.	Psychological preparation for young adults traveling abroad. Locke, Susan A.; Feinsod, Fred M.; Adolescence, Vol 17(68), Win 1982 pp. 815-819.	Unrelated
131.	Psychosomatic study of fifty stuttering children: Round table: I. Social, physical and psychiatric findings. Despert, J. Louise; American Journal of Orthopsychiatry, Vol 16(1), Jan, 1946 pp. 100-113.	Unrelated
132.	Psychosomatic study of fifty stuttering children: Round Table: IV. Rorschach study. Krugman, Morris; American Journal of Orthopsychiatry, Vol 16(1), Jan, 1946 pp. 127-133.	Unrelated
133.	Relationships between intolerance of uncertainty and eating disorder symptomatology in a mixed non-clinical, sub-clinical, and clinical eating disordered population. Stewart, Maria-Christina; Dissertation Abstracts International: Section B: The Sciences and Engineering, Vol 70(10-B) pp. 6569.	Unrelated
134.	Sildenafil treatment of paroxetine-induced anorgasmia in a woman. Ashton, Adam Keller; The American Journal of Psychiatry, Vol 156(5), May, 1999 pp. 800.	Unrelated
135.	Strategies for the control of unwanted thoughts in adolescents: The Adolescent Thought Control Questionnaire (TCQ-A). Whiting, Sara E.; May, Anna C.; Rudy, Brittany M.; Davis, Thompson E. III; Journal of Psychopathology and Behavioral Assessment, Vol 36(2), Jun, 2014 pp. 276-287.	Unrelated
136.	The factor structure of the Anorexia Nervosa Inventory for self-rating in a population-based sample and derivation of a shortened form. Rathner,	Unrelated

	Günther; Rainer, Barbara; European Archives of Psychiatry and Clinical Neuroscience, Vol 248(4), 1998 pp. 171-179.	
137.	The influence of obsessive compulsive (OC) behaviors on school functioning and help seeking intentions of high school students. Pertuit, Terry Lynn; Dissertation Abstracts International Section A: Humanities and Social Sciences, Vol 70(11-A) pp. 4193.	Unrelated
138.	The measurement of the obsessive-compulsive personality. Gibb, Gerald D.; Bailey, James R.; Best, Randall H.; Lambirth, Thomas T.; Educational and Psychological Measurement, Vol 43(4), Win 1983 pp. 1233-1238.	Unrelated
139.	The prevalence and effects of child sexual abuse in a poor, rural community in El Salvador: A retrospective study of women after 12 years of civil war. Barthauer, Linda M.; Leventhal, John M.; Child Abuse & Neglect, Vol 23(11), Nov, 1999 pp. 1117-1126.	Unrelated
140.	The usefulness of the Brief Symptom Inventory in the neuropsychological evaluation of traumatic brain injury. Slaughter, James; Johnstone, George; Petroski, Greg; Flax, Julia; Brain Injury, Vol 13(2), Feb, 1999 pp. 125-130.	Unrelated
141.	Trichotillomania: A phenomenological study. Chauhan, Sheelandra; Jain, R. K.; Dhir, G. G.; Indian Journal of Clinical Psychology, Vol 12(2), Sep, 1985 pp. 47-50.	Unrelated
142.	Understanding workaholism: A review of causes and counseling approaches. Seybold, Karen Colapietro; Salomone, Paul R.; Journal of Counseling & Development, Vol 73(1), Sep-Oct, 1994 pp. 4-9.	Review
143.	Use of a behavior-based personality inventory in aviation selection. Lambirth, Thomas T.; Gibb, Gerald D.; Alcorn, John D.; Educational and Psychological Measurement, Vol 46(4), Win 1986 pp. 973-978.	Unrelated
144.	Multifactorial aspects of eating disorders. Banaś, Anna; Januszkiewicz-Grabias, Aloiza; Radziwiłłowicz, Piotr; Psychiatria Polska, Vol 32(2), Mar-Apr, 1998 pp. 165-175.	Lumping
145.	A comparison of psychiatric comorbid symptomology between adolescents with restrictive/avoidant food intake disorder,anorexia nervosa and atypical anorexia nervosa.Wilson, D.;Krishnamorthy,G.; Mendes,R. A.;Withington, T.;Dalton, M.;Loxton, N. J.;European Eating Disorders Review,Vol 33(4),Jul, 2025, pp. https://doi.org/10.1002/erv.70014	Unrelated
146.	Sudden onset disordered eating behaviors and appetite issues in a local clinical cohort of children with pediatric acute-onset neuropsychiatric syndrome (PANS). Kapphahn,C.;Peet, B.; Gao, J.; Chan,A.; Farhadian, B.; Ma, M.; Silverman, M.; Tran, P.; Schlenk, N.; Thienemann, M.;Frankovich, J.; International Journal of Eating Disorders, Vol 58(3), Mar, 2025, pp.	Unrelated
147.	VPS13A disease: Bridging motor dysfunction and psychiatric symptoms – A case report. Simões, G.; Felgueiras, H.; Gomes, A. I.; Silva, R.; Malaquias, M. J.; Psychiatry Research Case Reports, Vol 4(1), Jun, 2025, pp.	Unrelated
148.	Treating obsessive compulsive disorder in adolescents and adults with Down syndrome: Results from a scoping rapid review. Fodstad, J. C.; Russell, R.; Bullington, M.; Jones, L. B.; Iticovici, M.; Meudt, E.; Journal of	Unrelated

	Autism and Developmental Disorders, Vol 55(5), May, 2025, pp. 1745–1753.	
149.	Self–other distinction and schizotypy: Affect sharing and alexithymia in the prediction of socially anxious and avoidant traits. Eddy, C. M.; Personality Disorders: Theory, Research, and Treatment, Vol 16(2), Mar, 2025, pp. 137–147.	Unrelated
150.	Borrelli DF, Dell UL, Barcaccia B, Tonna M, Dar R. The Italian validation of the sense of agency scale in a non-clinical sample: psychometric properties and associations with anxiety, depression, obsessive-compulsive, and psychotic symptoms. Current Psychology. 2026;45(1):1-12. doi: 10.1007/s12144-025-08695-x	Unrelated
151.	Esmaeili-Anamagh B, Mikaeili N. Journal of Psychology. Journal of Psychology. 2025;29(3):548-558. Accessed January 8, 2026.	Unrelated
152.	Halaç E, Ermis C, Gundogan N, et al. Neurocognitive and behavioral characteristics of ADHD with cognitive disengagement syndrome and specific learning disorder. Applied Neuropsychology: Child. September 2025. doi: 10.1080/21622965.2025.2565430	Duplicate
153.	Blažev D. Biological, Behavioural, Psychological and Social Predictors of Orthorexia Nervosa among Young Women in Croatia. Društvena Istraživanja. 2025;34(1):87-110. doi: 10.5559/di.34.1.05	Unrelated
154.	Kucukterzi AS, Ludlow AK, Gutierrez R, Fineberg NA, Gale TM. Pathological eating patterns in adults displaying obsessive-compulsive symptoms: A scoping review. European Eating Disorders Review. December 2025. doi: 10.1002/erv.70071	Duplicate
155.	Ricci F, Valentini CP, Torales J, et al. When imagination turns into disorder: the case of maladaptive daydreaming. International Review of Psychiatry. 2025;37(6/7):706-718. doi: 10.1080/09540261.2025.2562185	Duplicate
	Search from SCOPUS	
1.	Fong A., Friedlander R., Richardson A., Allen K., Zhang Q.Characteristics of children with autism and unspecified intellectual developmental disorder (intellectual disability) presenting with severe self-injurious behaviours (2024), 70 (3), pp. 518 - 529	Unrelated
2.	Gupta N., Gupta M.Off-label psychopharmacological interventions for autism spectrum disorders: Strategic pathways for clinicians (2024), 29 (1), pp. 10 - 25	Unrelated
3.	Keski-Rahkonen A.Eating disorders:etiology,risk factors,and suggestions for prevention(2024),37 (6),pp.381 - 387	No OCD
4.	Manwaring J.L., Blalock D.V., Rienecke R.D., Le Grange D., Mehler P.S. A descriptive study of treatment-seeking adults with avoidant/restrictive	Included

	food intake disorder at residential and inpatient levels of care (2024), 32 (1), pp. 13 - 28	
5.	Donini L.M.,Barrada J.R.,Barthels F.,Dunn T.M.,Babeau C.,Brytek-Matera A.,Cena H.,Stroebele-Benschop N.,Todisco P.,Vacca M.,Valente M.,Varga M.,Zagaria A.,Zickgraf H.F.,Lombardo C.A consensus document on definition and diagnostic criteria for orthorexia nervosa(2022), 27 (8),pp.3695 – 3711	Unrelated
6.	Zickgraf H.F., Ellis J.M., Essayli J.H. Disentangling orthorexia nervosa from healthy eating and other eating disorder symptoms: Relationships with clinical impairment, comorbidity, and self-reported food choices (2019), 134, pp. 40 - 49	Unrelated
7.	Hornberger L.L., Lane M.A. Identification and management of eating disorders in children and adolescents (2021),147 (1),art. no. e2020040279	No OCD
8.	Zickgraf H.F.,Murray H.B.,Kratz H.E.,Franklin M.E. Characteristics of outpatients diagnosed with the selective/neophobic presentation of avoidant/restrictive food intake disorder(2019), 52 (4), pp.367 - 377	Included
9.	Himmerich H., Bentley J., Kan C., Treasure J.Genetic risk factors for eating disorders: an update and insights into pathophysiology (2019), 9	Unrelated
10.	Oevreboe T.H.,Ivarsson A.,Sundgot-Borgen J.,Knudsen A.K.S.,Reneflot A.,Pensgaard A.M.Mental health problems in elite sport: the difference in the distribution of mental distress and mental disorders among a sample of Norwegian elite athletes (2023),9 (3),art.no.001538	Unrelated
11.	Lange C.R.A., Ekedahl Fjertorp H., Holmer R., Wijk E., Wallin U.Long-term follow-up study of low-weight avoidant restrictive food intake disorder compared with childhood-onset anorexia nervosa: Psychiatric and occupational outcome in 56 patients(2019), 52 (4), pp. 435 - 438	No OCD
12.	Medical Management of Restrictive Eating Disorders in Adolescents and Young Adults(2022), 71 (5),pp. 648 - 654	No OCD
13.	Richson B.N., Zickgraf H.F.Lifetime and current mental health based on avoidant/restrictive food intake disorder history versus other eating disorder history in the Healthy Minds Study(2024), 57 (10), pp. 1999 - 2005	Included (duplicate)
14.	Scott M., Leppanen J., Allen M., Jarrold C., Sedgewick F.Longitudinal Analysis of Mental Health in Autistic University Students Across an Academic Year (2023),53 (3),pp.1107-1116	Unrelated
15.	Zickgraf H.F.Treatment of pathologic healthy eating (orthorexia nervosa)(2019),pp.21-40 DOI: 10.1016/B978-0-12-816563-8.00002-4	Unrelated
16.	Kauer J., Pelchat M.L., Rozin P., Zickgraf H.F.Adult picky eating. Phenomenology, taste sensitivity, and psychological correlates (2015), 90, pp. 219 - 228	No ARFID
17.	Wald E.R., Eickhoff J., Flood G.E., Heinz M.V., Liu D., Agrawal A., Morse R.P., Raney V.M., Veerapandiyan A., Madan J.C.Estimate of the incidence of	Unrelated

	PANDAS and PANS in 3 primary care populations (2023), 11, art. no. 1170379	
18.	Kennedy S.M., Tonarely N.A., Halliday E., Ehrenreich-May J. A Person-Centered Approach to Understanding Heterogeneity of Youth Receiving Transdiagnostic Treatment for Emotional Disorders (2022), 90 (3), pp. 234 - 245	Unrelated
19.	Maertens C., Couturier J., Grant C., Johnson N.Fear of vomiting and low body weight in two pediatric patients: Diagnostic challenges (2017), 26 (1), pp. 59 – 63	Unrelated
20.	Kambanis P.E., Kuhnle M.C., Wons O.B., Jo J.H., Keshishian A.C., Hauser K., Becker K.R., Franko D.L., Misra M., Micali N., Lawson E.A., Eddy K.T., Thomas J.J.Prevalence and correlates of psychiatric comorbidities in children and adolescents with full and subthreshold avoidant/restrictive food intake disorder (2020), 53 (2), pp. 256 - 265	Included
21.	Fucà E., Guerrera S., Valeri G., Casula L., Novello R.L., Menghini D., Vicari S.Psychiatric Comorbidities in Children and Adolescents with High-Functioning Autism Spectrum Disorder: A Study on Prevalence, Distribution and Clinical Features in an Italian Sample (2023), 12 (2), art. no. 677	Unrelated
22.	Katzman D.K., Guimond T., Spettigue W., Agostino H., Couturier J., Norris M.L. Classification of Children and Adolescents With Avoidant/ Restrictive Food Intake Disorder (2022), 150 (3), art. no. e2022057494	Included
23.	Selles R.R., Wu M.S., Novoa J.C., Zepeda-Burgos R.M., Guttfreund D., McBride N.M., McKay D., Storch E.A. Prevalence, severity, and clinical correlates of food neophobia in Salvadorian youth (2021), 85 (1), pp. 42 - 58	Unrelated
24.	Bryson A.E., Scipioni A.M., Essayli J.H., Mahoney J.R., Ornstein R.M. Outcomes of low-weight patients with avoidant/restrictive food intake disorder and anorexia nervosa at long-term follow-up after treatment in a partial hospitalization program for eating disorders(2018), 51 (5), pp. 470 – 474	Included
25.	Deng Z., Liu J., Gong H., Cai X., Xiao H., Gao W. Psychiatric disorders associated with PCSK9 inhibitors: A real-world, pharmacovigilance study (2024), 30 (4), art.no.e14522 DOI: 10.1111/cns.14522	Unrelated
26.	Tanldlr C., Hergüner S. Mirtazapine for Choking Phobia: Report of a Pediatric Case (2015), 25 (8), pp. 659 – 660 DOI: 10.1089/cap.2015.0145	Unrelated
27.	Zickgraf H.F., Franklin M.E., Rozin P. Adult picky eaters with symptoms of avoidant/restrictive food intake disorder: Comparable distress and comorbidity but different eating behaviors compared to those with disordered eating symptoms (2016), 4 (1), art. no. 26	Included (duplicate)
28.	Biswas T., Sinha A., Abhijita B., Mishra S., Padhy S.K.Pediatric Acute Onset Neuropsychiatric Syndrome Presenting with Atypical Eating Disorder: A Case Report (2022), 18 (4),pp.364 - 366	Case Report
29.	Kambanis P.E., Harshman S.G., Kuhnle M.C., Kahn D.L., Dreier M.J., Hauser K., Slattery M., Becker K.R., Breithaupt L., Misra M., Micali N., Lawson E.A., Eddy	No OCD

	K.T.,Thomas J.J.Differential comorbidity profiles in avoidant/restrictive food intake disorder and anorexia nervosa: Does age play a role? (2022), 55 (10), pp. 1397 – 1403	
30.	Paslakis G., Woodside B., Katzman D.K. Editorial: Recent advances in diagnosis and treatment of comorbid conditions in eating disorders (2023), 13, art. no. 1117831	Lumping
31.	Velimirović M., Robison M., Abber S., Duffy A., Rienecke R.D., Manwaring J., Blalock D.V., Riddle M., Mehler P.S., Joiner T.E. Anxiety, Obsessive-Compulsive, and Depressive Symptom Presentation and Change Throughout Routine Eating Disorder Treatment (2024)	Included (duplicate)
32.	Herre M., Yang K.H., Francois D. Food for thought: Dangerous weight loss in an older adult (2022),21(4),pp.47–52 DOI:10.12788/cp.0224	Unrelated
33.	Toufexis M.D., Hommer R., Gerardi D.M., Grant P., Rothschild L., D'Souza P., Williams K., Leckman J., Swedo S.E., Murphy T.K. Disordered eating and food restrictions in children with PANDAS/PANS (2015), 25 (1), pp. 48 - 56	Unrelated
34.	Ogden J. Eating disorders in the pandemic (2022), 26 (2), pp. 16 – 17 DOI: 10.1002/pnp.744	Unrelated
35.	Sanzari C.M., Hormes J.M. U.S. health professionals' perspectives on orthorexia nervosa: clinical utility, measurement and diagnosis, and perceived influence of sociocultural factors (2023),28(1),art.no.31	Unrelated
36.	Kreipe R.E., Palomaki A. Beyond picky eating: Avoidant/restrictive food intake disorder (2012), 14(4),pp.421-431	No OCD
37.	Görmez A., Kılıç A., Kırpınar İ. Avoidant/Restrictive Food Intake Disorder: An Adult Case Responding to Cognitive Behavioral Therapy (2018), 17 (6), pp. 443 - 452	No OCD
38.	Selles R.R., Best J.R., Lu C., Soreni N., Farrell L.J., Mathieu S., Stewart S.E. Challenges of insight assessment in pediatric obsessive-compulsive disorder: Initial results and clinical considerations from a measure development study (2023), 36, art. no. 100774	Unrelated
39.	Suganya M., Prabakar S., Mahadeva Rao U.S. Cognitive Behavioural Therapy in Children and Adolescents (2022), 15 (3), pp. 1330 - 1336	Unrelated
40.	Marchetto, C.; Criscuolo, M.; Croci, I.; Bucci, M. E.; Caramadre, A.; Castiglioni, M. C.; Vicari, S.; Zanna, V. Family functioning and eating psychopathology in developmental restrictive eating disorders after Covid-19 lockdown. Eating and Weight Disorders, Vol 30(1), 2025, art. no. 36. https://doi.org/10.1007/s40519-025-01749-w	Unrelated
41.	Chen, L.-C.; Bai, Y.-M.; Tsai, S.-J.; Cheng, C.-M.; Chen, M.-H. Eating disorders, psychiatric comorbidities, and suicide. Journal of Affective Disorders, Vol 382, 2025, pp. 290–295. https://doi.org/10.1016/j.jad.2025.04.090	Unrelated
42.	Towards a mechanistic framework for understanding early-onset restrictive eating disorders. Nature Mental Health, Vol 3(7), 2025, pp. 761–762. https://doi.org/10.1038/s44220-025-00458-8	Unrelated

43.	Łojek, P.; Rzeszutek, M. PANS and PANDAS – symptoms beyond OCD and tics – a systematic review. Journal of Psychiatric Research, Vol 187, 2025, pp. 144–153. https://doi.org/10.1016/j.jpsychires.2025.05.001	Unrelated
44.	Kapphahn, C.; Peet, B.; Gao, J.; Chan, A.; Farhadian, B.; Ma, M.; Silverman, M.; Tran, P.; Schlenk, N.; Thienemann, M.; Frankovich, J. Sudden onset disordered eating behaviors and appetite issues in a local clinical cohort of children with pediatric acute-onset neuropsychiatric syndrome (PANS). International Journal of Eating Disorders*,* Vol 58(7), 2025, pp. 1219–1232. https://doi.org/10.1002/eat.24388	Unrelated
45.	Moreau, C. A.; Ayrolles, A.; Ching, C. R. K.; Bonicel, R.; Mathieu, A.; Stordeur, C.; El Khantour, C.; Bergeret, P.; Traut, N.; Tran, L.; Germanaud, D.; Alison, M.; Elmaleh-Bergès, M.; Ehrlich, S.; Thompson, P. M.; Bourgeron, T.; Delorme, R. Neuroimaging insights into brain mechanisms of early-onset restrictive eating disorders. Nature Mental Health*,* Vol 3(7), 2025, pp. 780–788. https://doi.org/10.1038/s44220-025-00447-x	Unrelated
46.	Califano, M.; Pruccoli, J.; Cavallino, O.; Lenzi, A.; Parmeggiani, A. Psychopathological comorbidities in children and adolescents with feeding and eating disorders: An Italian clinical study. Pediatrics*,* Vol 17(3), 2025, art. no. 61. https://doi.org/10.3390/pediatric17030061	No OCD
47.	Bauman, T.; Voderholzer, U. Pharmacotherapy of eating disorders—An update [Pharmakotherapie der Essstörungen – ein Update]. Der Nervenarzt, Vol 96(3), 2025, pp. 230–237. https://doi.org/10.1007/s00115-025-01804-y	Unrelated
48.	Li, R.; Radhakrishnan, V. A case of avoidant/restrictive food intake disorder in an adult with schizophrenia and obsessive-compulsive disorder. Journal of the Academy of Consultation-Liaison Psychiatry*,* Vol 66(3), 2025, pp. 268–269. https://doi.org/10.1016/j.jaclp.2025.01.005	Case Report
49.	Velimirović, M.; Robison, M.; Abber, S.; Duffy, A.; Rienecke, R. D.; Manwaring, J.; Blalock, D. V.; Riddle, M.; Mehler, P. S.; Joiner, T. E. Anxiety, obsessive-compulsive, and depressive symptom presentation and change throughout routine eating disorder treatment. European Eating Disorders Review*,* Vol 33(3), 2025, pp. 490–502. https://doi.org/10.1002/erv.3160	Included (duplicate)
50.	Wronski, M.-L.; Kuja-Halkola, R.; Hedlund, E.; Martini, M. I.; Lichtenstein, P.; Lundström, S.; Larsson, H.; Taylor, M. J.; Micali, N.; Bulik, C. M.; Dinkler, L. Mental and somatic conditions in children with the broad avoidant restrictive food intake disorder phenotype. JAMA Pediatrics*,* Vol 179(4), 2025, pp. 428–437. https://doi.org/10.1001/jamapediatrics.2024.6065	Included (duplicate)
51.	Richson, B. N.; Schaefer, L. M.; Becker, K. R.; Murray, M. F.; Romano, K. A.; Anderson, L. M.; Wonderlich, S. A.; Thomas, J. J. Empirical approaches to the classification of avoidant/restrictive food intake disorder. International Journal of Eating Disorders*,* Vol 58(2), 2025, pp. 291–307. https://doi.org/10.1002/eat.24341	No OCD
52.	Schmidt, U. H.; Claudino, A.; Fernández-Aranda, F.; Giel, K. E.; Griffiths, J.; Hay, P. J.; Kim, Y.-R.; Marshall, J.; Micali, N.; Monteleone, A. M.; Nakazato, M.; Steinglass, J.; Wade, T. D.; Wonderlich, S.; Zipfel, S.; Allen, K. L.; Sharpe, H. The current clinical approach to feeding and eating disorders aimed to increase personalization of management. World Psychiatry*,* Vol 24(1), 2025, pp. 4–31. https://doi.org/10.1002/wps.21263	Unrelated
53.	McDuffee, N. S.; Forster, A. A.; Shah, K.; Easterday, P. Factitious disorder in the context of abuse in an adolescent: Importance of a multidisciplinary	Unrelated

	approach. Psychiatric Case Reports, Vol 27(1), 2025, art. no. 24cr03832. https://doi.org/10.4088/PCC.24cr03832	
54.	Wilson, D.; Krishnamorthy, G.; Mendes, R. A.; Withington, T.; Dalton, M.; Loxton, N. J. A comparison of psychiatric comorbid symptomology between adolescents with restrictive/avoidant food intake disorder, anorexia nervosa and atypical anorexia nervosa. European Eating Disorders Review, 2025, [in stampa]. https://doi.org/10.1002/erv.70014	No OCD
55.	Brambilla, P. Highlights of the September 2025 issue (2025) Minerva Psychiatry, 66 (3), pp. 127 - 128, Cited 0 times. DOI: 10.23736/S2724-6612.25.02680-6	Unrelated
56.	Kennedy, A., Scodellaro, S., Verstegen, R.H.J., Cohn, I. Pharmacogenetic Testing of Children and Adolescents with Mental Health Conditions: Real-World Experiences (2025) Pharmaceuticals, 18 (8), art. no. 1170, Cited 0 times. DOI: 10.3390/ph18081170	Unrelated
57.	Kucukterzi-Ali, S., Ludlow, A.K., Gutierrez, R., Fineberg, N.A., Gale, T.M. Pathological Eating Patterns in Adults Displaying Obsessive-Compulsive Symptoms: A Scoping Review (2025) European Eating Disorders Review, Cited 0 times. DOI: 10.1002/erv.70071	Duplicate
	Other sources	
1.	Sader M, Harris HA, Waiter GD, Jackson MC, Voortman T, Jansen PW, Williams JHG. Prevalence and Characterization of Avoidant Restrictive Food Intake Disorder in a Pediatric Population. JAACAP Open. 2023 May 24;1:116-127. Doi: 10.1016/j.jaacop.2023.05.001.	Included

Supplementary Table S2. Risk of bias assessment

Study	Risk of bias level
Zickgraf et al., 2016	Low
Wronski et al., 2024	Medium
Velimirović et al., 2024	Low
Richson et al., 2024	Medium

Sader et al., 2023	Low
Zickgraf et al., 2019	Low
Manwaring et al., 2024	Low
Bryson et al.,2018	Medium
Kambanis et al.,2020	Low
